# Risk factors associated with the development of seizures among adult patients treated with ertapenem: A matched case-control study

**DOI:** 10.1371/journal.pone.0182046

**Published:** 2017-07-31

**Authors:** Yi-Chien Lee, Yun-Jhong Huang, Miao-Chiu Hung, Sheng-Che Hung, Chih-Yen Hsiao, Hui-Ling Cho, Li-Fen Lai, Show-Hwa Tong, Jann-Tay Wang

**Affiliations:** 1 Department of Internal Medicine, Ditmanson Medical Foundation Chia-Yi Christian Hospital, Chia-Yi, Taiwan; 2 Department of Sports Management, Chia Nan University of Pharmacy and Science, Tainan, Taiwan; 3 Department of Colorectal Surgery, Ditmanson Medical Foundation Chia-Yi Christian Hospital, Chia-Yi, Taiwan; 4 Department of Pediatrics, Taipei Veterans General Hospital, Taipei, Taiwan; 5 Department of Radiology, Taipei Veterans General Hospital, Taipei, Taiwan; 6 School of Medicine, National Yang-Ming University, Taipei, Taiwan; 7 Department of Hospital and Health Care Administration, Chia Nan University of Pharmacy and Science, Tainan, Taiwan; 8 Department of Nursing, Ditmanson Medical Foundation Chia-Yi Christian Hospital, Chia-Yi, Taiwan; 9 Department of Pharmacy, Ditmanson Medical Foundation Chia-Yi Christian Hospital, Chia-Yi, Taiwan; 10 Department of Internal Medicine, National Taiwan University Hospital, Taipei, Taiwan; Radboud Universiteit, NETHERLANDS

## Abstract

**Objective:**

The purpose of this study is to compare the characteristics of those ertapenem-treated adult patients with and without development of seizures, and identify the associated factors for the development of seizures.

**Methods:**

This retrospective study was conducted at Chia-Yi Christian Hospital from January 2012 to December 2014. Patients developing seizures during their ertapenem treatment course were identified as case patients. Those without seizures who had received ertapenem for at least five days were considered as the pool of control patients. For each case patient, four matched patients from the control pool were randomly selected as the final control group, based on age, gender, and the date of ertapenem prescription.

**Results:**

A total of 1706 ertapenem-treated patients were identified, 33 (1.9%) individuals developed seizures with the enrollment of 132 matched control patients. Among these 33 patients, the average age was 79.3 ± 7.5 years, and 20 (60.6%) were male. The mean Charlson co-morbidity score was 4.5 ± 2.4, and the first episode of seizure happened 3.3 ± 2.6 days after receiving ertapenem. In multivariate logistic regression analysis, the independent predictors associated with the development of ertapenem-associated seizures were old stroke (OR, 14.36; 95% CI, 4.38–47.02; p < 0.0001), undergoing brain images within one year prior to the admission (OR, 5.73; 95% CI, 1.78–18.43; p = 0.0034), low hemoglobin level (OR, 3.88; 95% CI, 1.28–12.75; p = 0.0165) and low platelet count (OR, 4,94; 95% CI, 1.56–15.68; p = 0.0067) at presentations, and protective factors against the development of seizures were heart failure (OR, 0.04; 95% CI, 0.00–0.63; p = 0.0222), concomitant use of steroids (OR, 0.19; 95% CI, 0.05–0.77; p = 0.0201), or antiplatelet agents (OR, 0.12; 95% CI, 0.02–0.63, p = 0.0123) with ertapenem.

**Conclusions:**

The development of ertapenem-associated seizures may occur more frequently and much earlier due to its widespread use in treating drug-resistant pathogens, especially when these pathogens emerged worldwide.Our study would help physician to estimate the risk of developing seizure among patients receiving ertapenem.

## Introduction

Carbapenems possess greatest broad-spectrum activity against gram-positive and gram-negative aerobic and anaerobic bacteria. Among them, imipenem, meropenem, ertapenem, and doripenem have been approved for clinical use in various countries, including Taiwan [1]. They have been proven to be effective against serious infections, including bloodstream infections, nosocomial pneumonia, intra-abdominal infections, and complicated urinary tract infections [2–5].

Although good tolerability has been observed, the most common side effects of carbapenems were gastrointestinal tract upset with an estimated incidence of around 1% to 5% [6–9]. In addition, seizures (abnormal excessive or synchronous electrical discharge in the brain with clinical presentations of involuntary motor movements) have been associated with all carbapenems [6–9]. The mechanism of carbapenem-associated seizures has been thought to be directly associated with the resemblance of the β-lactam ring with the conformation of the γ-aminobutyric acid (GABA) neurotransmitter and antagonism at the receptor site [10,11]. Although such an adverse reaction happens rarely, uncontrolled seizures could lead to major injury or even increased mortality due to impaired level of consciousness or loss of motor control [12].

In general, the incidence of seizures associated with carbapenems is as follows, in order of decreasing frequency: Meropenem (0.7%) [6], ertapenem (0.5%) [7], and imipenem (0.4%) [8], according to FDA-approved labeling; but compared to other carbapenems, the highest seizure rate has occurred with the use of imipenem (3.8%), in clinical trials [13]. However, in a meta-analysis study directly comparing imipenem and meropenem, no difference existed in the rate of seizures in pooled OR analyses [14]. Among those imipenem-treated patients, important risk factors for seizures identified were high dose therapy (> 25 mg/kg), renal impairment, and preexisting neurologic disorders [15].

Nevertheless, only a few case reports or case series discussing the association between ertapenem use and seizures have been published [16–18,19–23]. Thus, we designed a case-control study to investigate the factors associated with seizures in those adult patients receiving ertapenem therapy to provide much safer prescription of the antimicrobial agent.

## Methods

### Ethics statement

This study was reviewed and approved by the Institutional Review Board of Chia-Yi Christian Hospital (CYCH), a 1000-bed regional teaching hospital in southern Taiwan (Approval # CYCH-IRB-105066). The IRB waived informed consent due to the retrospective study design and the research posing no more than minimal risk. All primary data were collected according to procedures outlined in epidemiology guidelines to strengthen the reporting of observational studies.

### Study design and settings

In CYCH, ertapenem was introduced in 2008. Physicians became aware of the growing number of patients developing seizures associated with the use of ertapenem thereafter. Accordingly, this retrospective 1:4 case-control study was carried out at CYCH to identify the associated factors for seizures in patients receiving ertapenem. The list of all patients aged 18 years or above admitted at CYCH and receiving at least one dose of ertapenem to treat their bacterial infections between January 2012 and December 2014 was retrieved from the computerized database of the Department of Pharmacy. For patients in the list, case patients were defined as those developing a seizure during the course of ertapenem treatment. The pool of control patients were those who did not develop seizures while receiving ertapenem and who were administered at least five days of ertapenem, as previous case series revealed that the first seizure episode occurred a mean of 6.7 days after start of ertapenem therapy [24]. For each case patient, 4 non-seizure controls with matching criteria such as sex, age (± 5 years), and the date of ertapenem prescription (± 30 days) were randomly selected from the control pool.

### Data collection

Electronic and written medical records for all enrolled patients were reviewed. A standardized case report form was utilized to collect information on their demographics and clinical characteristics such as age, gender, underlying diseases, undergoing brain images due to neurologic symptoms or signs within one year prior to this admission, previous use of medications within one month prior to this hospitalization, initial vital signs and laboratory data before the use of ertapenem, intensive care unit (ICU) admission, infection syndromes treated by ertapenem, dosage and treatment duration of ertapenem, concomitant medications during the treatment course of ertapenem, length of hospital stay, and in-hospital mortality. Underlying diseases included neurologic comorbidities, such as old stroke, parkinsonism, dementia and epilepsy. For patients with seizures, the onset date of seizures, results of computed tomography (CT) scan of brain performed for the seizure, electroencephalograph (EEG) performed for the seizure, and management of seizures were also recorded.

### Definitions

Seizures were defined as any abnormal motor movements with or without dyscognitive features, including focal and generalized type [12], and every episode of seizure was documented in medical records by the primary care physicians. Patients were considered to have chronic kidney disease if their baseline estimated glomerular filtration rate (eGFR) calculated by Modification of Diet in Renal Disease (MDRD) formula was less than 45 ml/min/1.73 m^2^. Utilization of some specific medications was categorized into the following: calcium channel blockers including amlodipine, verapamil, and diltiazem; angiotensin-converting enzyme inhibitors or angiotensin receptor blockers including captopril, valsartan, and losartan; diuretics including spironolactone, hydrochlorothiazide, trichlormethiazide, and furosemide; beta-blockers including propranolol, bisoprolol, carvedilol, atenolol, and labetalol; alpha-blockers including terazosin, and doxazosin; nitrates including isosorbide mononitrate, and isosorbide dinitrate; antimicrobial agents including levofloxacin, ceftibuten, amoxicillin, cefuroxime, amoxicillin/clavulanate, azithromycin, trimethoprim/sulfamethoxazole, ciprofloxacin, clarithromycin, norfloxacin, ceftazidime, fluconazole, vancomycin, aminoglycoside, metronidazole, cefotaxime, ampicillin, colimycin, oxacillin, micafungin, daptomycin, and fosfomycin; antiplatelet agents including aspirin, clopidogrel, dipyridamole and ticlopidine; prokinetic agents including metoclopramide, domperidone, and mosapride; sedative-hypnotics including fludiazepam, midazolam, diazepam, lorazepam, clonazepam, alprazolam, zopiclone, estazolam, and zolpidem; opioids including morphine, tramadol, meperidine, and fentanyl; non-steroid anti-inflammatory drugs including ketorolac, diclofenac, etoricoxib, and celecoxib. During the study period, the recommended ertapenem doses were as described in the package insert of ertapenem [7], i.e., the dose of ertapenem given was one gram once a day in those patients with normal renal function and 500 mg daily if their creatinine clearance ≦ 30 ml/min/1.73m^2^, calculated by Cockcroft & Gault equation.

### Statistical analysis

Continuous variables were described as means and standard deviations, and analyzed using a Mann-Whitney *U*-test. Categorical variables were expressed as frequency and proportions, and compared with a chi-square test or Fisher’s exact test if the expected number was less than or equal to 10. Factors associated with the development of seizure were identified using a conditional logistic regression model. All potential variables associated were tested using univariate analysis first. Factors with a *p* value less than 0.2 were then included in the multivariate analysis using the backward stepwise method. All tests were two-tailed and a *p*-value < 0.05 was considered statistically significant. Statistical analyses were performed using SPSS 17.0 software (International Business Machines Corp., Armonk, NY, USA).

## Results

### Baseline characteristics of enrolled patients

**Fig 1:**
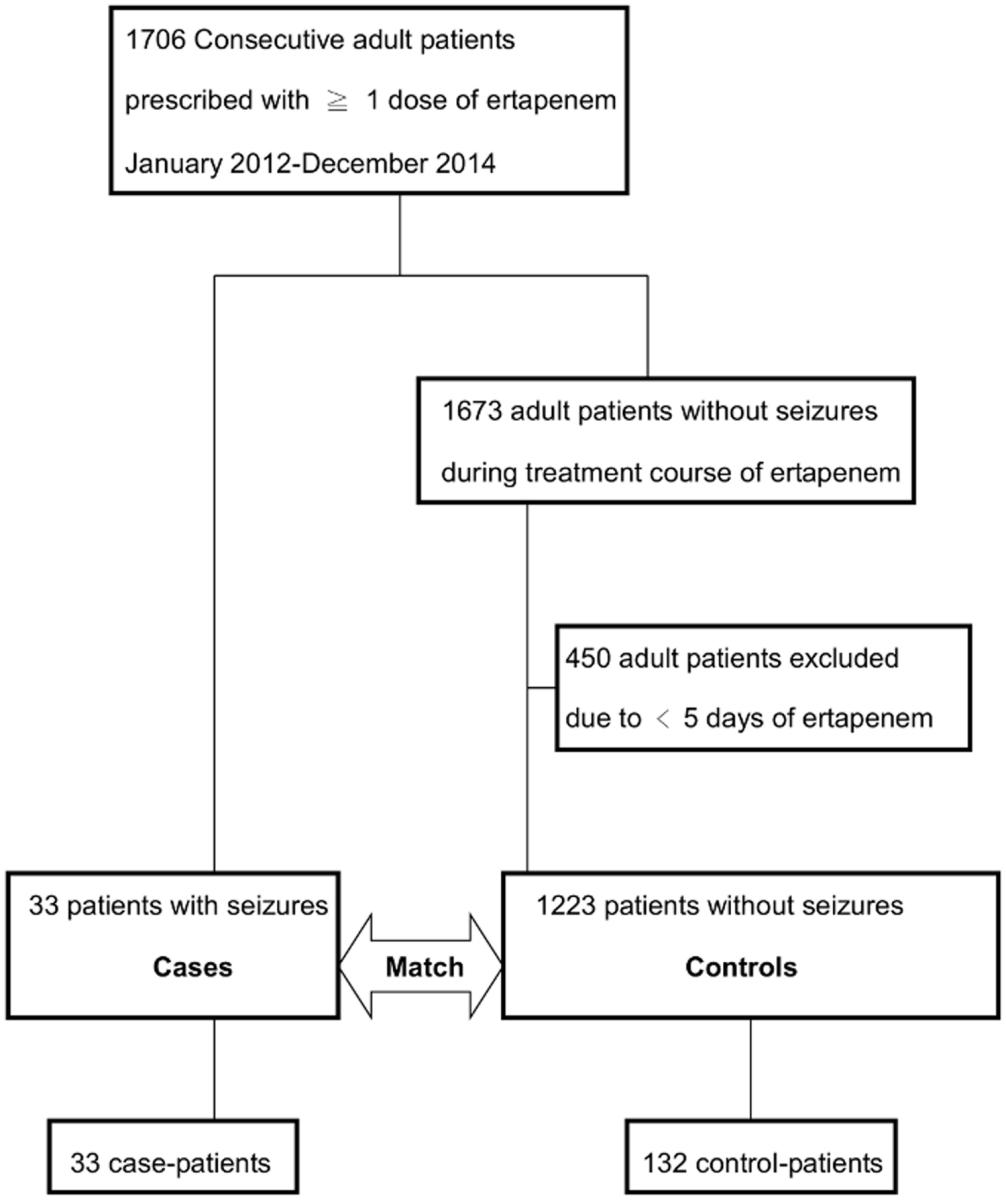
Flow chart of participants with or without seizures included in the analysis.

During the 3-year study period, a total of 1706 adult patients were prescribed with at least one dose of ertapenem therapy at CYCH, and seizures developed in 33 patients (1.9%, case patients) following the administration of ertapenem. Among the 1673 patients without seizures (designated as the pool of controls), 450 patients were excluded because they received less than five days of ertapenem. Out of the remaining 1223 potential controls, 132 were matched by sex, age, and the date of prescription of ertapenem to the 33 cases patients to survey the associated factors of attacks of seizures (Fig 1). The baseline characteristics of the 165 enrolled patients are summarized in Tables 1 and 2 (S1 File). Compared with control patients, case patients were more likely to have a history of old stroke (either hemorrhage or infarct), epilepsy, and receiving brain image studies within one year prior to this admission, and a higher Charlson comorbidity score. Concerning prior exposure to various medications, fewer case patients were prescribed with sedative-hypnotics than with the controls (p = 0.044), but no significant difference existed within other categories of drugs. Additionally, a lower proportion of case patients received concomitant use of steroids with ertapenem, but no other categories of medications concomitantly used with ertapenem differed significantly. Lower blood glucose, and more patients with anemia (hemoglobin < 11 g/dl) or thrombocytopenia (platelets < 150k /mm^3^) were observed in the case patients.

**Table 1:** Clinical characteristics in matched pairs (1:4) of those ertapenem-treated patients with or without development of seizures.

Variables	Patients with seizures	Patients without siezures	p-value
No. of patients	33	132	-
Gender (male)	20 (60.6)	80 (60.6)	-
Age	79.3±7.5	78.8±7.7	0.7116^†^
Hypertension	23 (69.7)	78 (59.1)	0.2634^¥^
Diabetes mellitus	13 (39.4)	58 (43.9)	0.6371^¥^
Old stroke	22 (66.7)	32 (24.2)	<.0001^¥^
Cerebrovascular disease type			<.0001^¥^
Intracerebral hemorrhage	10 (30.3)	9 (6.8)	
Ischemic infarction	12 (36.4)	23 (17.4)	
Parkinsonism	5 (15.2)	15 (11.4)	0.5556^¥^
Dementia	8 (24.2)	24 (18.2)	0.4309^¥^
Epilepsy	4 (12.1)	3 (2.3)	0.0302^¥^
Heart failure	1 (3.0)	18 (13.6)	0.1264^¥^
Coronary artery disease	2 (6.1)	23 (17.4)	0.1034^¥^
Peripheral arterial occlusive disease	3 (9.1)	4 (3.0)	0.1437^¥^
Cancer	12 (36.4)	30 (22.7)	0.1077^¥^
Gout	3 (9.1)	14 (10.6)	-
Liver cirrhosis	6 (18.2)	14 (10.6)	0.2402^¥^
Chronic obstructive pulmonary disease	1 (3.0)	17 (12.9)	0.1277^¥^
Peptic ulcer disease	11 (33.3)	45 (34.1)	0.9345^¥^
Chronic kidney disease	9 (27.3)	31 (23.5)	0.6497^¥^
End stage renal disease	2 (6.1)	11 (8.3)	-
Charlson score	4.5±2.4	3.4±2.5	0.0244^†^
Brain image within one year prior to this admission	16 (48.5)	28 (21.2)	0.0015^¥^
Intracranial hemorrhage	2	1	0.5920^¥^
Infarction	14	23	0.9919^¥^
Hydrocephalus	9	12	0.3977^¥^
Chronic subdural effusion	0	1	-
Meningioma	0	1	-
Initial vital signs before use of ertapenem			
Conscious change	8 (24.2)	20 (15.2)	0.2134^¥^
Systolic blood pressure	136.8±28.3	132.6±31.9	0.4886^†^
Diastolic blood pressure	76.4±18.4	72.7±16.8	0.2653^†^
Heart rate	99.0±17.4	94.6±19.5	0.2410^†^
Body temperature	37.3±1.3	37.4±1.1	0.7166^†^
Respiratory rate	23.2±4.5	21.7±7.4	0.1433^†^
Initial lab data before use of ertapenem			
Blood sugar	147.7±58.7	184.5±107.9	0.0184^†^
White blood count	8520.1±6007.1	10818.8±5986.3	0.0503^†^
White blood count < 4000 or > 11000	18 (54.6)	66 (50.0)	0.6404^¥^
Hemoglobin	10.5±4.0	10.8±2.2	0.7137^†^
Hemoglobin < 11 g/dl	26 (78.8)	67 (50.8)	0.0037^¥^
Platelet	178.1±102.1	205.0±103.3	0.1873^†^
Platelet < 150k/mm^3^	16 (50.0)	40 (30.3)	0.0350^¥^
C-reactive protein	10.5±8.3	9.4±8.7	0.6071^†^
Aspartate aminotransferase	79.1±106.5	46.7±53.3	0.1794^†^
Alanine aminotransferase	79.0±147.1	50.5±130.5	0.3374^†^
Blood urea nitrogen	39.0±33.0	35.4±31.3	0.5767^†^
Creatinine	1.9±2.1	1.9±1.8	0.9885^†^
Creatinine > 1.4	10 (32.3)	53 (41.7)	0.3341^¥^
Sodium	135.9±12.1	136.1±7.4	0.9035^†^
Potassium	3.7±0.8	3.8±0.6	0.4548^†^
Baseline creatinine	1.6±2.3	1.2±1.0	0.2864^†^
Intensive care unit admission	10 (30.3)	37 (28.0)	0.7958^¥^
Recommended dose	20 (60.6)	98 (74.2)	0.1206^¥^
Duration of hospitalization, days	24.2±17.1	19.3±12.4	0.1250^†^
In-hospital mortality	11 (33.3)	14 (10.6)	0.0011^¥^
The mean time-to-onset of seizures, days	3.3±2.6	-	
Computed tomography scan of brain	17 (51.5)	-	
Electroencephalography	24 (72.7)	-	
Medications for control of seizures (31)			
Benzodiazepines	23 (74.2)	-	
Antiepileptic drugs	30 (96.8)	-	

^†^ Student t;

^¥^ Chi-Square test.

**Table 2:** Prior and concomitant medications used in those ertapenem-treated patients with or without development of seizures.

Variables	Patients with seizures (33)	Patients without siezures(132)	p-value
Previous drug history within one month	29 (87.9)	98 (74.2)	0.0961^¥^
Calcium channel blocker	15 (45.5)	43 (32.6)	0.1658^¥^
ACEI or ARB	4 (12.1)	22 (16.7)	0.5215^¥^
Diuretics	6 (18.2)	26 (19.7)	0.8439^¥^
Beta blocker	2 (6.1)	15 (11.4)	0.5286^¥^
Alpha blocker	1 (3.0)	10 (7.6)	0.6954^¥^
Harnalidge	4 (12.1)	12 (9.1)	0.5293^¥^
Nitrate	1 (3.0)	10 (7.6)	0.6954^¥^
Statin	3 (9.1)	9 (6.8)	0.7081^¥^
Antibiotics	7 (21.2)	19 (14.4)	0.3363^¥^
H2-blocker	9 (27.3)	33 (25.0)	0.7886^¥^
Proton pump inhibitors	4 (12.1)	23 (17.4)	0.4614^¥^
Steroid	3 (9.1)	17 (12.9)	0.7673^¥^
Antiplatelet agents	5 (15.2)	31 (23.5)	0.2999^¥^
Prokinetic agents	9 (27.3)	29 (22.0)	0.5175^¥^
Drugs for peripheral vascular disorder	3 (9.1)	16 (12.1)	0.7677^¥^
Sedative/hypnotics	2 (6.1)	28 (21.2)	0.0435^¥^
Concomitant drugs with ertapenem	33 (100.0)	130 (98.5)	-
Proton pump inhibitors	17 (51.5)	52 (39.4)	0.2067^¥^
H2-blockers	11 (33.3)	49 (37.1)	0.6858^¥^
Prokinetic agents	12 (36.4)	41 (31.1)	0.5595^¥^
Harnalidge	2 (6.1)	15 (11.4)	0.5286^¥^
Albumin	6 (18.2)	27 (20.5)	0.7703^¥^
Opioids	6 (18.2)	32 (24.2)	0.4595^¥^
Non-steroid anti-inflammatory drug	0 (0.0)	9 (6.8)	0.2067^¥^
Steroid	4 (12.1)	41 (31.1)	0.0289^¥^
Inhaled bronchodilators	14 (42.4)	47 (35.6)	0.4680^¥^
Beta-blockers	2 (6.1)	18 (13.6)	0.3711^¥^
Diuretics	10 (30.3)	46 (34.9)	0.6218^¥^
Calcium channel blockers	10 (30.3)	51 (38.6)	0.3751^¥^
Statin	2 (6.1)	6 (4.6)	0.6609^¥^
Nitrate	3 (9.1)	10 (7.6)	0.7249^¥^
Cordarone	1 (3.0)	10 (7.6)	0.6954^¥^
Knowful	4 (12.1)	10 (7.6)	0.4826^¥^
Antiplatelet agents	3 (9.1)	28 (21.2)	0.1108^¥^
Drugs for peripheral vascular disorder	2 (6.1)	8 (6.1)	-
Transamine	7 (21.2)	18 (13.6)	0.2776^¥^
Sedative/hypnotics	5 (15.2)	25 (18.9)	0.6138^¥^
Antibiotics	6 (18.2)	32 (24.2)	0.4595^¥^
Contrast medium	2 (6.1)	20 (15.2)	0.2529^¥^

^¥^ Chi-Square test.

ACEI: angiotensin converting enzyme inhibitor; ARB: angiotensin receptor blocker.

Ertapenem was mainly used to treat patients with a diagnosis of UTI, followed by pneumonia, and application of this antimicrobial agent showed no significant difference between case and control patients. Around 60% (20/33) of case patients and approximately three-fourths of control patients received the recommended doses, and the proportion of ICU admission initially was similar (30.3% vs. 28.0%). In this study, 11 case subjects and 29 control patients received normal dose based on their renal function. Otherwise, lower dose than recommended was prescribed in 2 cases and 5 controls, respectively. No association with subtherapeutic dose and the development of seizure existed (p = 0.85). No patients in the case or control group received supratherapeutic dose. Concerning treatment outcome, longer length of hospitalization and higher crude in-hospital mortality were noted in the case group (p = 0.0011). The median duration of ertapenem therapy was 4 days (IQR, 2–6), and 8 days (IQR, 7–11) in case subjects and control patients, respectively.

The first episode of seizure among case patients occurred within 3.3 ± 2.6 days after initiation of ertapenem treatment. Of these 33 patients with the development of seizures, 23 individuals presented as generalized tonic clonic seizure, 9 as focal seizure, and one as absence seizure. CT scans of the brain and EEGs were arranged for 17 (51.5%) and 24 (72.7%) patients, respectively. The CT findings included infarction in 15, hydrocephalus in 9, normal results in 2, and hemorrhage in one; and the EEGs showed diffuse cortical dysfunction in 22, epileptogenic discharge in 5, normal results in one, and generalized sharp waves in one. Only two patients did not take medications used to control the seizures. Of 31 patients receiving medical therapy for their ertapenem-associated seizure, 23 (74.2%) cases took benzodiazepines, including diazepam, midazolam and lorazepam, and 30 (96.8%) individuals were prescribed with at least one of the following antiepileptic drugs: phenytoin (21 patients, 67.7%), valproic acid (11, 35.5%), levetiracetam (9, 29%), and topiramate (1, 3.2%). Ten (30.3%) patients were admitted to the ICU initially, and the mean of hospital stay and the crude in-hospital mortality rate were 24.2 ± 17.1 days and 33.3%, respectively.

### Factors associated with the development of seizures among patients treated with ertapenem

In univariate analysis using a conditional logistic regression model, old stroke, epilepsy, undergoing brain images within one year prior to this admission, a higher Charlson co-morbidity score, lower hemoglobin level, lower platelet count, and no concomitant use of steroids with ertapenem were associated with the development of seizures during ertapenem treatment (Table 3). Multivariate analysis identified old stroke (OR, 14.36; 95% CI, 4.38–47.02; p < 0.0001), undergoing brain images within one year prior to this admission (OR, 5.73; 95% CI, 1.78–18.43; p = 0.0034), lower hemoglobin level (OR, 3.88; 95% CI, 1.28–12.75; p = 0.0165), and lower platelet count (OR, 4,94; 95% CI, 1.56–15.68; p = 0.0067) as significant predictors for the development of seizure. However, heart failure (OR, 0.04; 95% CI, 0.00–0.63; p = 0.0222), use of steroids (OR, 0.19; 95% CI, 0.05–0.77; p = 0.0201) or antiplatelet agents concurrentwith ertapenem (OR, 0.12; 95% CI, 0.02–0.63, p = 0.0123) were independently protective factors.

**Table 3:** Multivariate analysis for factors associated with the development of seizures in patients treated with ertapenem.

Covariate	Univariate			Multivariate		
	OR (95% CI)		P Value	OR (95% CI)		P Value
Old stroke	6.17	(2.71–14.04)	<.0001	14.36	(4.38–47.02)	<.0001
Epilepsy	5.84	(1.25–27.30)	0.0249			
Heart failure	0.20	(0.03–1.55)	0.1228	0.04	(0.00–0.63)	0.0222
Coronary artery disease	0.31	(0.07–1.37)	0.1225			
peripheral arterial occlusive disease	3.17	(0.68–14.81)	0.1423			
Malignancy	1.94	(0.86–4.37)	0.1124			
chronic obstructive pulmonary disease	0.21	(0.03–1.66)	0.1393			
Charlson score	1.18	(1.02–1.37)	0.0280			
Brain image within one year before this admission	3.46	(1.56–7.69)	0.0022	5.73	(1.78–18.43)	0.0034
Prior use of calcium channel blocker within 1 month	1.72	(0.79–3.73)	0.1698			
Prior use of sedative or hypnotics within 1 month	0.24	(0.05–1.07)	0.0609			
Initial hemoglobin < 11 g/dl	3.58	(1.46–8.79)	0.0055	3.88	(1.28–11.75)	0.0165
Initial platelet < 150k/mm^3^	2.29	(1.05–5.01)	0.0384	4.94	(1.56–15.68)	0.0067
Recommended dose	0.54	(0.24–1.19)	0.1250			
Concomitant use of steroid	0.31	(0.10.0.93)	0.0369	0.19	(0.05–0.77)	0.0201
Concomitant use of antiplatelet agents	0.37	(0.11–1.31)	0.1240	0.12	(0.02–0.63)	0.0123
Concomitant use of contrast medium	0.36	(0.08–1.63)	0.1868			

## Discussion

To our knowledge, this study was the first matched case-control study to compare the clinical characteristics of patients with or without development of seizures during the treatment course of ertapenem, and investigate the predictors for the development of seizures among these patients. The proportion of patients developing seizures among those individuals treated with ertapenem was about 1.9% in the present study, and the interval from the initiation of ertapenem to the onset of seizure was 3.3 ± 2.6 days. The independent predictors associated with the development of seizures were old stroke, undergoing brain images within one year previous to this admission, lower hemoglobin level, and lower platelet count; and heart failure, concomitant use of steroid or antiplatelet agent with ertapenem were protective factors from seizures.

Several preceding clinical trials have reported that the proportion developing seizure among those patients treated with ertapenem was around 0.1% [24] to 0.5% [25], much lower than that in our study (1.9%). This might be because patients enrolled in our study were older (mean age, 71 vs. <60 years), and old patients at higher risk for ertapenem-associated seizures has been illustrated previously [19,24,26]. In addition, the interval from the administration of ertapenem to the onset of seizures was shorter than that noted in prior clinical trials [16–18]. As we understand it, multiple complicated co-morbidities were excluded in previous clinical trials, such as chronic kidney disease [16], thrombocytopenia [16,17], or malignancy [17]; all of these were noted among our studied patients. That might be the reason why earlier development of seizures occurred in our ertapenem-treated patients, but the actual mechanism for latency from symptom onset to diagnosis of seizures should be further surveyed.

Several prior studies have demonstrated that pre-existing central nervous system (CNS) disorders, including old cerebrovascular accidents (either thrombotic or embolic events), were risk factors for the development of seizures among ertapenem-treated patients [20–23]. One possible hypothesis was that ertapenem might act as a mediator lowering the seizure threshold in patients with previous neurological comorbidities [22]. Another deduction has been that damage to the blood-brain barrier with a subsequently higher concentration of ertapenem in the brain tissue, observed in patients with a history of cerebrovascular events, would result in development of antibiotic-related seizures [27]. Our study echoed this finding, and the requirement of neuroimages for neurologic manifestations within one year prior to this hospitalization, which might imply pre-existing CNS disorders, also increased the risk of seizures.

If, clinically, some surrogate markers or laboratory parameters could be recognized to predict the potential of developing antibiotic-associated seizures, it would be helpful for physicians to be alert to, detect, and then manage them as soon as possible. Among pediatric patients, iron deficiency anemia (hemoglobin value < 11 g/dl), as a significant risk factor for febrile seizures has been clarified in some case-control studies [28–30]. Low hemoglobin might impair oxygen delivery to brain tissue, which in turn might contribute to the development of brain dysfunction, including the development of seizures. However, the detailed mechanisms need further investigation.

Berggren et al. observed that thrombocytopenia was more common among those alcohol-dependent individuals with development of seizures compared to those without alcohol-related seizures: Numminen et al. showed thrombocytopenia correlated with the onset of brain infarction in alcoholics; and Kim et al reported that 62.5% of patients had thrombocytopenia when they developed seizures [31–33]. All these findings supported that thrombocytopenia is possibly one of the underlying mechanisms contributing to the development of seizures. In addition, thrombocytopenia might be a surrogate marker of poor general condition. And patients with poorer general condition might thus more easily develop seizures while they receive ertapenem.

The immunomodulatory or anti-inflammatory properties of corticosteroids made them effective for treatment of seizures or epilepsy, with the first report published in 1942 [34–36]. Corticosteroids can suppress the corticotropin-releasing hormone levels in the CNS and then lower the neuronal excitability; also, they can affect GABA_A_ receptors or enhance the action of neurosteroids [37]. Thus, it is reasonable that concomitant use of steroids with ertapenem would protect those patients from developing seizures, as shown in our study.

Several recent clinical reviews have emphasized activation of the inflammatory process that is probably involved with the occurrence of epilepsy [38–40]. Among atherothrombotic diseases, such as myocardial infarction or ischemic stroke, platelets contribute to vascular inflammation, so use of antiplatelet agents would manifest an anti-inflammatory effect by attenuating the release of inflammatory mediators [41]. Furthermore, acetylsalicylic acid (ASA) in high concentrations has had direct neuroprotective effects in animal models [42], and even ASA itself or in combination with anti-epileptic drugs lowers the incidence of seizures [43–45]. Therefore, the protective effects of antiplatelet agents occurring concurrently with the use of ertapenem from the development of seizures as demonstrated in our results is reasonable, but the actual platelet-mediated inflammatory pathways in the CNS correlated with seizures caused by ertapenem should be surveyed in the future.

Heart failure has been well recognized as a complex clinical syndrome resulting from structural or functional impairment of ventricular filling or ejection of blood. As it occurs, subsequent compensatory mechanisms that activate the renin-angiotensin-aldosterone system would start to restore adequate cardiac output [46,47]. Xu B et al. have demonstrated that angiotensin II in the central nervous system potentiates GABA release, which in turn increases the seizure threshold [48]. This might partially explain why patients with heart failure would be less likely to develop seizure while receiving ertapenem.

There were limitations in our study. First, it was retrospective and conducted at a single center, so unavoidable bias, confounding, and missing data, and generalization cautiously would be anticipated. In particular, history of old stroke or epilepsy could be only documented by medical records. It was difficult to verify the information about if these patients with a history of old stroke or epilepsy had acute symptomatic seizures in their life previously or had a lesion due to a stroke visible on brain image studies, which might lead to imprecise estimation of our statistics. Additionally, the history of febrile or afebrile seizures in patients or families might be a risk factor for such drug induced seizures, but acquiring the information was not feasible in our retrospective study. Second, drug concentrations of ertapenem in the serum and cerebral tissues were not calculated, so it was difficult to judge the impact of drug levels on the development of seizures. Finally, some patients receiving brain image studies within one year prior to this admission, but we cannot clarify whether these lesions were epileptogenic and would therefore directly cause subsequent episodes of seizure or not. Nonetheless, our study results remain useful for clinical physicians in dealing with ertapenem-treated patients because we have identified several factors associated with the development of seizure among these patients. This should alert clinicians to detect and manage seizure disorders as soon as possible.

In conclusion, with the widespread use of ertapenem due to the increasing rate of resistant pathogens, ertapenem-associated seizures, in the elderly particularly, would be expected to occur more frequently, and even much earlier. Our study results may help clinicians to estimate the risk of developing seizures when ertapenem is prescribed to treat bacterial infections in those patients, and to prescribe ertapenem more safely.

## Supporting information

S1 File Dataset of 165 ertapenem-treated patients with or without development of seizures. (PDF)
