# Supplementary material for: Risk factors associated with the development of seizures among adult patients treated with ertapenem: A matched case-control study
Source: PLoS One. 2017 Jul 31;12(7):e0182046. doi: 10.1371/journal.pone.0182046 (PMC5536326; doi:10.1371/journal.pone.0182046)
Supplement: S1 File — (PDF) [file pone.0182046.s001.pdf]

index	case	gender	age	Hypertension	Diabetes mellitus	Old stroke	Cerebrovascular disease type intracerebral hemorrhage	Cerebrovascular disease type ischemic infarction	Parkinsonism	Dementia	Epilepsy	Heart failure	Coronary artery disease	Peripheral arterial occlusive disease	Cancer	Gout
1	1	0	76	1	0	1	1	0	0	0	0	0	0	0	0	0
1	0	0	72	1	0	0	0	0	0	0	0	0	0	0	0	0
1	0	0	73	1	0	0	0	0	0	0	0	0	0	0	1	0
1	0	0	73	0	1	0	0	0	0	0	0	0	1	0	1	0
1	0	0	79	1	0	0	0	0	0	1	0	0	0	0	0	0
2	1	0	69	1	0	1	0	1	0	0	1	0	0	0	1	0
2	0	0	70	0	0	0	0	0	0	0	0	0	0	0	0	1
2	0	0	75	1	0	0	0	0	0	0	0	0	1	0	0	0
2	0	0	68	1	1	0	0	0	0	0	0	0	0	0	0	0
2	0	0	69	0	1	0	0	0	0	0	0	0	0	0	0	0
3	1	1	85	0	0	0	0	0	0	1	0	0	0	0	0	0
3	0	1	85	1	1	0	0	0	1	1	0	0	0	0	1	0
3	0	1	87	1	1	1	0	1	1	1	0	0	0	1	0	0
3	0	1	86	1	1	0	0	0	1	0	1	1	1	0	0	1
3	0	1	86	0	0	1	1	0	0	0	0	0	0	0	0	0
4	1	0	89	1	0	1	0	1	1	0	0	0	0	0	1	1
4	0	0	90	1	0	0	0	0	0	1	0	0	0	0	1	0
4	0	0	86	1	0	0	0	0	0	0	0	0	0	0	0	0
4	0	0	86	1	1	1	1	0	0	0	0	1	0	0	0	0
4	0	0	87	1	0	0	0	0	0	0	0	0	0	0	0	0
5	1	1	84	0	0	0	0	0	0	0	0	0	0	0	1	0
5	0	1	83	1	0	0	0	0	0	0	0	0	0	0	0	0
5	0	1	86	1	0	0	0	0	0	1	0	0	0	0	0	1
5	0	1	83	1	0	0	0	0	0	0	0	0	0	0	1	0
5	0	1	83	0	0	0	0	0	0	0	0	0	0	0	1	0
6	1	1	87	1	1	0	0	0	0	0	0	0	0	0	1	0
6	0	1	82	0	0	1	0	1	0	0	0	0	0	0	0	0
6	0	1	91	0	0	1	1	0	0	0	0	0	0	0	0	0
6	0	1	86	0	0	0	0	0	0	0	0	0	1	1	0	0
6	0	1	90	0	1	1	0	1	0	0	0	0	0	0	0	0
7	1	1	80	0	0	0	0	0	0	1	0	0	0	0	0	0
7	0	1	82	0	0	0	0	0	0	0	0	0	0	0	0	0
7	0	1	81	1	0	0	0	0	0	0	0	0	1	0	0	0
7	0	1	83	0	0	1	1	0	0	0	0	0	0	0	0	0
7	0	1	75	1	0	0	0	0	0	0	0	0	0	0	0	0
8	1	1	81	1	1	1	1	0	0	0	0	0	0	0	1	0
8	0	1	83	0	1	0	0	0	0	0	0	1	0	0	0	1
8	0	1	86	0	0	0	0	0	0	0	0	0	0	0	0	0
8	0	1	86	0	0	0	0	0	1	0	0	0	0	0	0	0

8	0	1	78	0	0	0	0	0	0	0	0	0	0	0	1	0
9	1	1	85	1	0	1	0	1	0	0	0	0	0	1	0	0
9	0	1	83	0	0	0	0	0	0	0	0	0	0	0	1	0
9	0	1	75	1	0	1	0	1	0	0	0	0	0	0	0	0
9	0	1	79	1	0	0	0	0	0	0	0	0	0	0	0	1
9	0	1	82	0	1	0	0	0	0	0	0	1	1	0	0	1
10	1	1	73	0	0	1	1	0	0	0	0	0	0	0	0	0
10	0	1	69	0	0	0	0	0	0	0	0	0	0	0	0	0
10	0	1	71	0	1	0	0	0	0	0	0	0	0	0	0	0
10	0	1	72	1	0	1	0	1	0	0	0	0	0	0	0	0
10	0	1	70	1	0	1	1	0	0	0	0	0	0	0	0	0
11	1	0	76	1	0	1	0	1	0	0	0	0	0	0	0	0
11	0	0	81	1	1	1	0	1	0	0	1	0	0	0	0	0
11	0	0	72	1	1	0	0	0	0	0	0	0	0	0	1	0
11	0	0	79	0	0	0	0	0	0	0	0	0	0	0	1	0
11	0	0	75	1	1	1	0	1	0	0	0	0	0	0	1	0
12	1	0	66	1	1	1	0	1	0	0	0	0	1	0	0	0
12	0	0	70	1	0	0	0	0	0	0	0	0	0	0	0	0
12	0	0	63	0	1	0	0	0	0	0	0	0	0	0	0	0
12	0	0	64	1	0	0	0	0	0	0	0	0	0	0	1	0
12	0	0	66	0	1	0	0	0	0	0	0	0	0	0	1	0
13	1	1	81	1	1	1	1	0	1	0	1	0	0	0	0	0
13	0	1	86	1	0	0	0	0	0	0	0	0	0	0	0	1
13	0	1	78	1	1	0	0	0	0	0	0	0	1	0	0	0
13	0	1	82	0	0	0	0	0	0	0	0	0	1	0	0	0
13	0	1	81	0	0	0	0	0	0	0	0	0	1	0	0	1
14	1	0	77	1	0	0	0	0	0	0	0	0	0	0	1	0
14	0	0	81	1	1	1	1	0	0	1	0	1	0	0	1	0
14	0	0	80	1	0	0	0	0	0	0	0	0	1	0	0	1
14	0	0	75	1	1	1	0	1	0	0	0	0	0	0	0	0
14	0	0	77	0	0	0	0	0	0	0	0	0	0	0	0	0
15	1	0	97	0	0	0	0	0	0	1	0	0	0	1	0	0
15	0	0	88	1	1	0	0	0	0	0	0	1	0	0	0	0
15	0	0	87	1	0	1	0	1	0	0	0	0	0	0	0	0
15	0	0	86	0	1	0	0	0	0	0	0	1	1	0	0	0
15	0	0	89	1	0	0	0	0	0	0	0	0	0	0	1	0
16	1	0	89	1	1	1	0	1	0	0	0	0	0	1	0	1
16	0	0	84	1	0	1	0	1	0	0	0	1	0	0	0	0
16	0	0	91	1	1	1	0	1	0	1	0	0	0	0	0	0
16	0	0	90	0	0	0	0	0	0	0	0	0	0	0	0	0
16	0	0	87	1	1	0	0	0	1	1	0	0	0	0	0	0
17	1	0	71	1	0	0	0	0	0	0	0	0	0	0	1	0

17	0	0	68	0	0	0	0	0	0	0	0	0	0	0	0	0
17	0	0	75	0	1	0	0	0	0	0	0	0	0	0	1	0
17	0	0	72	1	1	0	0	0	0	0	0	0	0	0	0	0
17	0	0	65	0	0	0	0	0	0	0	0	0	0	0	0	0
18	1	1	62	1	0	0	0	0	0	0	0	0	0	0	1	0
18	0	1	60	0	1	0	0	0	0	0	0	0	0	0	1	0
18	0	1	61	1	1	0	0	0	0	0	0	0	0	0	1	0
18	0	1	58	1	1	0	0	0	0	0	1	0	0	0	0	1
18	0	1	54	1	0	0	0	0	0	0	0	0	0	0	0	0
19	1	0	81	1	0	1	1	0	0	0	0	0	0	0	0	0
19	0	0	82	0	0	0	0	0	0	0	0	0	0	0	0	0
19	0	0	80	0	0	0	0	0	0	0	0	0	0	0	1	0
19	0	0	86	1	0	1	0	1	0	1	0	0	1	0	1	0
19	0	0	78	1	0	1	0	1	0	0	0	1	0	0	0	0
20	1	0	85	1	0	0	0	0	1	0	0	0	0	0	0	1
20	0	0	88	1	1	1	1	0	0	1	0	0	0	0	0	0
20	0	0	84	1	1	1	0	1	0	0	0	1	0	0	0	0
20	0	0	88	0	1	0	0	0	0	1	0	0	0	0	0	0
20	0	0	83	0	1	0	0	0	0	0	0	0	0	0	1	0
21	1	1	81	1	1	1	1	0	0	0	0	0	0	0	0	0
21	0	1	77	0	0	1	1	0	0	0	0	0	0	0	1	0
21	0	1	78	0	0	0	0	0	0	0	0	1	1	0	0	0
21	0	1	81	0	0	1	1	0	1	0	0	0	0	0	0	0
21	0	1	83	1	1	0	0	0	0	0	0	0	0	0	0	0
22	1	1	76	0	0	0	0	0	0	1	0	0	0	0	1	0
22	0	1	78	1	0	1	0	1	1	1	0	0	0	0	0	0
22	0	1	75	1	1	0	0	0	0	0	0	0	0	0	1	0
22	0	1	74	1	0	0	0	0	1	0	0	0	0	0	0	0
22	0	1	72	0	0	0	0	0	0	1	0	0	0	0	1	0
23	1	1	82	0	1	1	0	1	0	0	0	0	0	0	0	0
23	0	1	84	0	0	0	0	0	0	1	0	0	0	0	0	0
23	0	1	84	0	1	0	0	0	0	0	0	0	0	0	0	0
23	0	1	85	0	0	0	0	0	0	0	0	0	0	0	1	0
23	0	1	82	1	0	1	0	1	0	0	0	0	0	0	0	0
24	1	1	80	0	0	1	1	0	0	0	0	0	0	0	0	0
24	0	1	79	0	1	0	0	0	1	1	0	0	0	0	0	0
24	0	1	84	0	0	0	0	0	0	0	0	0	0	0	0	0
24	0	1	78	0	1	0	0	0	1	1	0	0	0	0	0	0
24	0	1	77	1	0	0	0	0	0	0	0	0	0	0	0	1
25	1	1	78	0	0	1	1	0	1	1	0	0	0	0	1	0
25	0	1	78	0	0	0	0	0	1	0	0	0	0	0	0	0
25	0	1	76	1	0	0	0	0	0	0	0	0	0	0	1	0

25	0	1	74	1	1	0	0	0	1	0	0	1	1	1	0	1
25	0	1	76	0	0	0	0	0	0	0	0	0	0	0	1	0
26	1	1	77	1	0	0	0	0	0	0	0	0	0	0	1	0
26	0	1	81	1	1	0	0	0	0	0	0	0	0	0	1	0
26	0	1	80	1	1	0	0	0	0	0	0	0	0	0	0	0
26	0	1	76	0	0	0	0	0	0	0	0	0	0	0	0	0
26	0	1	79	1	1	1	0	1	0	1	0	0	0	0	0	0
27	1	1	74	1	1	1	0	1	0	0	0	0	0	0	0	0
27	0	1	79	0	0	0	0	0	0	0	0	0	0	0	0	0
27	0	1	78	1	1	0	0	0	1	1	0	0	1	0	0	0
27	0	1	79	1	0	0	0	0	0	0	0	0	0	0	0	0
27	0	1	79	1	0	0	0	0	0	0	0	0	0	0	0	0
28	1	1	69	1	1	1	0	1	1	1	0	0	0	0	0	0
28	0	1	67	0	0	0	0	0	0	0	0	1	1	0	0	0
28	0	1	64	1	1	1	0	1	0	0	0	0	1	0	0	0
28	0	1	65	1	1	0	0	0	0	0	0	1	1	0	0	0
28	0	1	68	1	1	0	0	0	0	0	0	0	0	0	0	0
29	1	1	75	1	1	1	0	1	0	0	0	0	1	0	0	0
29	0	1	70	1	1	0	0	0	0	0	0	0	0	0	0	0
29	0	1	72	0	1	0	0	0	0	0	0	0	1	0	0	0
29	0	1	71	1	1	0	0	0	0	0	0	0	0	0	0	0
29	0	1	80	1	1	0	0	0	0	0	0	0	0	0	0	0
30	1	1	85	1	1	1	1	0	0	1	1	0	0	0	0	0
30	0	1	81	1	1	0	0	0	0	0	0	0	0	1	0	0
30	0	1	88	0	1	1	0	1	0	1	0	0	0	0	0	0
30	0	1	86	1	0	1	0	1	1	1	0	1	0	0	0	0
30	0	1	80	1	0	0	0	0	0	0	0	0	0	0	1	0
31	1	0	84	0	1	1	0	1	0	0	1	1	0	0	0	0
31	0	0	89	1	1	0	0	0	1	0	0	1	0	0	0	0
31	0	0	82	1	0	0	0	0	0	0	0	0	0	0	0	0
31	0	0	85	1	1	0	0	0	0	1	0	0	0	0	0	1
31	0	0	88	1	1	0	0	0	0	1	0	0	0	0	0	0
32	1	1	72	1	0	1	1	0	0	0	0	0	0	0	1	0
32	0	1	70	1	1	1	0	1	0	0	0	0	1	0	0	0
32	0	1	75	1	1	0	0	0	0	0	0	0	0	0	0	0
32	0	1	77	1	0	0	0	0	0	0	0	1	1	0	0	1
32	0	1	75	0	0	0	0	0	0	0	0	0	0	0	0	0
33	1	0	90	1	1	1	0	1	0	1	0	0	0	0	0	0
33	0	0	91	1	1	1	0	1	0	0	0	0	1	0	1	0
33	0	0	92	0	0	0	0	0	0	1	0	0	0	0	0	0
33	0	0	83	1	1	1	0	1	0	1	0	0	0	0	0	0
33	0	0	85	1	0	0	0	0	0	0	0	1	1	0	0	0

Liver cirrhosis	Chronic obstructive pulmonary disease	Peptic ulcer disease	Chronic kidney disease	End stage renal disease	Charlson score	Brain image within one year prior to this	Brain image Intracranial hemorrhage	Brain image Infarction	Brain image Hydrocephalus	Brain image Chronic subdural effusion	Brain image Meningioma	Initial vital signs conscious change	Systolic blood pressure
1	0	1	0	0	6	1	1	0	0	0	0	1	142
0	0	0	0	0	0	0	0	0	0	0	0	0	182
0	0	1	1	1	11	0	0	0	0	0	0	0	117
0	0	1	0	0	4	0	0	0	0	0	0	0	140
0	0	0	1	1	3	0	0	0	0	0	0	0	157
0	0	1	1	1	10	1	0	1	0	0	0	0	164
0	0	0	0	0	1	0	0	0	0	0	0	0	68
0	0	0	1	1	3	0	0	0	0	0	0	0	118
1	0	0	0	0	4	0	0	0	0	0	0	0	118
0	0	0	0	0	2	0	0	0	0	0	0	0	161
0	1	0	0	0	2	1	0	1	0	0	0	0	144
1	0	0	0	0	6	1	0	0	1	0	0	0	161
0	1	0	0	0	6	0	0	0	0	0	0	0	130
0	0	0	0	0	4	1	0	1	1	0	0	1	96
0	0	0	0	0	2	0	0	0	0	0	0	0	129
0	0	1	1	1	7	1	0	1	0	0	0	0	115
0	0	0	0	0	7	0	0	0	0	0	0	0	82
1	0	1	0	0	2	0	0	0	0	0	0	0	119
0	0	0	0	0	5	1	0	0	1	0	0	1	134
0	0	1	1	1	3	0	0	0	0	0	0	1	138
1	0	1	0	0	8	1	0	1	1	0	0	0	128
0	0	1	1	0	1	0	0	0	0	0	0	0	170
0	0	0	1	0	1	1	0	1	1	0	0	0	156
0	0	1	0	0	7	1	1	0	0	0	0	0	161
0	0	0	0	0	2	0	0	0	0	0	0	0	102
0	0	0	1	0	6	0	0	0	0	0	0	0	122
0	0	0	0	0	1	0	0	0	0	0	0	0	125
0	1	0	0	0	3	0	0	0	0	0	0	0	131
0	0	1	0	0	3	0	0	0	0	0	0	1	70
0	0	0	0	0	3	0	0	0	0	0	0	0	149
0	0	0	0	0	1	1	0	1	1	0	0	0	124
0	0	0	0	0	0	0	0	0	0	0	0	0	143
0	0	1	0	0	2	0	0	0	0	0	0	0	149
0	1	1	0	0	4	0	0	0	0	0	0	1	112
0	0	1	0	0	1	0	0	0	0	0	0	0	102
0	0	0	0	0	6	0	0	0	0	0	0	0	142
0	1	1	1	0	3	0	0	0	0	0	0	0	147
0	0	0	0	0	0	0	0	0	0	0	0	0	94
1	1	0	0	0	2	1	0	0	0	1	0	0	165

0	0	1	0	0	7	0	0	0	0	0	0	0	157
0	0	0	0	0	3	0	0	0	0	0	0	0	102
0	1	0	0	0	7	0	0	0	0	0	0	0	144
0	0	0	0	0	1	0	0	0	0	0	0	0	128
0	0	0	1	1	2	0	0	0	0	0	0	1	114
0	1	0	0	0	4	0	0	0	0	0	0	0	91
0	0	0	0	0	2	1	0	0	1	0	0	1	144
0	0	1	1	0	1	0	0	0	0	0	0	0	99
0	1	0	0	0	3	0	0	0	0	0	0	0	121
0	0	1	1	0	4	1	0	1	0	0	0	0	189
0	0	1	0	0	2	0	0	0	0	0	0	0	138
0	0	0	0	0	1	0	0	0	0	0	0	0	182
0	0	0	0	0	4	1	0	1	0	0	0	0	102
0	0	1	0	0	9	0	0	0	0	0	0	0	80
1	0	1	0	0	4	0	0	0	0	0	0	0	140
0	0	0	0	0	6	0	0	0	0	0	0	0	147
0	0	0	0	0	5	1	0	1	1	0	0	0	148
0	0	0	0	0	0	0	0	0	0	0	0	0	82
0	0	1	1	1	5	0	0	0	0	0	0	0	213
1	0	0	0	0	7	0	0	0	0	0	0	0	113
0	0	0	0	0	8	0	0	0	0	0	0	0	171
0	0	0	0	0	4	1	0	1	1	0	0	0	124
0	1	0	0	0	1	0	0	0	0	0	0	0	137
0	0	1	1	0	4	1	0	0	1	0	0	0	86
0	0	1	0	0	2	0	0	0	0	0	0	0	162
0	0	0	0	0	1	1	0	1	0	0	0	0	152
0	0	1	1	0	5	0	0	0	0	0	0	0	95
0	0	0	0	0	8	0	0	0	0	0	0	0	153
0	0	0	1	0	1	0	0	0	0	0	0	1	102
0	0	0	0	0	3	0	0	0	0	0	0	0	107
0	0	0	1	0	0	0	0	0	0	0	0	0	148
0	0	0	0	0	2	0	0	0	0	0	0	1	131
0	0	1	0	0	4	0	0	0	0	0	0	0	138
0	0	0	0	0	2	1	0	1	0	0	0	0	134
0	1	0	1	0	4	0	0	0	0	0	0	0	146
0	0	1	0	0	3	0	0	0	0	0	0	0	107
1	0	1	1	0	7	0	0	0	0	0	0	0	109
0	0	1	1	0	4	1	0	1	1	0	0	0	204
0	0	0	0	0	4	0	0	0	0	0	0	0	157
0	0	0	0	0	0	0	0	0	0	0	0	0	110
0	0	0	1	0	3	1	0	1	1	0	0	0	225
1	0	1	0	0	6	0	0	0	0	0	0	0	149

0	0	1	1	1	3	0	0	0	0	0	0	0	87
0	0	0	1	0	4	0	0	0	0	0	0	0	154
1	0	0	1	1	7	0	0	0	0	0	0	0	179
0	0	0	0	0	0	0	0	0	0	0	0	0	113
1	0	1	1	0	8	0	0	0	0	0	0	1	133
1	0	0	0	0	11	0	0	0	0	0	0	0	92
1	0	0	0	0	7	0	0	0	0	0	0	0	161
0	0	1	0	0	3	0	0	0	0	0	0	0	167
1	0	0	0	0	1	0	0	0	0	0	0	0	163
0	0	0	0	0	2	0	0	0	0	0	0	0	138
1	0	1	1	0	6	0	0	0	0	0	0	0	134
0	0	0	1	1	8	0	0	0	0	0	0	0	100
0	0	0	0	0	5	1	0	1	0	0	0	0	160
0	0	0	0	0	2	1	0	1	1	0	0	1	96
0	0	0	1	0	1	1	1	1	0	0	0	1	164
0	0	1	0	0	4	0	0	0	0	0	0	0	158
0	0	0	0	0	3	0	0	0	0	0	0	1	170
0	0	0	0	0	2	0	0	0	0	0	0	1	106
1	0	1	0	0	5	0	0	0	0	0	0	0	97
0	0	0	0	0	3	1	0	1	1	0	0	0	168
0	0	0	0	0	4	1	0	1	0	0	0	0	108
0	0	0	0	0	2	0	0	0	0	0	0	0	152
0	0	0	0	0	1	0	0	0	0	0	0	1	117
0	0	0	0	0	1	0	0	0	0	0	0	0	167
0	0	0	0	0	1	1	0	1	1	0	0	1	197
0	0	0	0	0	3	1	0	1	1	0	0	1	100
0	0	0	0	0	3	0	0	0	0	0	0	0	154
0	0	0	1	1	2	1	0	1	0	0	0	1	157
0	0	0	1	1	5	0	0	0	0	0	0	1	158
0	0	1	0	0	4	0	0	0	0	0	0	0	97
0	0	1	0	0	2	0	0	0	0	0	0	0	83
0	0	1	0	0	2	1	0	1	0	0	0	0	195
0	0	0	0	0	2	1	0	1	0	0	0	0	87
0	0	0	0	0	1	0	0	0	0	0	0	0	158
0	0	0	0	0	2	0	0	0	0	0	0	0	114
0	0	0	0	0	2	0	0	0	0	0	0	0	170
0	0	0	0	0	0	0	0	0	0	0	0	0	106
0	0	0	0	0	2	1	0	1	0	0	0	1	98
0	0	0	0	0	0	0	0	0	0	0	0	0	148
0	0	0	0	0	4	0	0	0	0	0	0	0	130
0	0	1	0	0	4	0	0	0	0	0	0	0	109
0	0	1	0	0	7	0	0	0	0	0	0	0	99

0	0	0	1	0	6	0	0	0	0	0	0	0	153
1	0	0	0	0	3	0	0	0	0	0	0	0	110
1	0	0	1	0	9	0	0	0	0	0	0	0	175
0	1	1	1	0	6	0	0	0	0	0	0	0	132
0	1	0	0	0	2	1	0	1	0	0	0	0	198
0	1	0	0	0	1	0	0	0	0	0	0	0	159
0	0	1	0	0	6	0	0	0	0	0	0	0	105
0	0	0	1	0	5	1	0	1	0	0	0	0	148
0	0	0	0	0	0	0	0	0	0	0	0	0	106
0	1	0	0	0	3	0	0	0	0	0	0	0	111
0	0	0	0	0	0	0	0	0	0	0	0	0	113
0	0	0	0	0	0	0	0	0	0	0	0	0	115
0	0	0	0	0	4	0	0	0	0	0	0	0	111
0	0	0	0	0	2	0	0	0	0	0	0	0	141
0	0	1	1	0	7	1	0	1	1	0	0	0	162
0	0	0	1	0	6	0	0	0	0	0	0	0	139
0	0	0	0	0	1	1	0	1	0	0	0	0	138
0	0	0	0	0	3	1	0	1	1	0	0	0	109
0	0	0	0	0	1	0	0	0	0	0	0	0	210
0	0	0	1	0	4	0	0	0	0	0	0	0	112
0	0	0	0	0	1	0	0	0	0	0	0	0	160
0	0	0	1	0	3	0	0	0	0	0	0	0	150
0	0	1	0	0	6	0	0	0	0	0	0	0	122
0	0	0	0	0	3	0	0	0	0	0	0	0	135
0	1	1	0	0	7	0	0	0	0	0	0	0	107
0	0	0	0	0	4	1	0	1	1	0	0	0	114
0	1	1	0	0	8	1	0	1	0	0	0	0	85
0	0	1	0	0	6	1	0	1	1	0	0	1	95
0	0	1	0	0	4	0	0	0	0	0	0	1	92
1	0	1	0	0	2	0	0	0	0	0	0	0	140
0	0	0	0	0	2	0	0	0	0	0	0	0	159
0	1	1	0	0	4	0	0	0	0	0	0	0	91
0	0	0	0	0	4	0	0	0	0	0	0	0	142
0	0	0	0	0	5	0	0	0	0	0	0	0	175
0	0	0	0	0	2	0	0	0	0	0	0	0	135
0	0	1	1	0	5	1	0	1	0	0	0	1	117
0	0	1	0	0	1	0	0	0	0	0	0	0	109
0	0	0	0	0	5	1	0	1	0	0	0	1	208
0	0	1	0	0	11	0	0	0	0	0	0	1	140
0	0	1	0	0	2	0	0	0	0	0	0	1	118
0	0	0	0	0	5	1	0	1	1	0	1	1	117
0	0	1	0	0	3	0	0	0	0	0	0	0	133

Diastolic blood pressure	Heart rate	Body temperature	Respiratory rate	Initial lab data Blood sugar	White blood count	White blood count < 4000 or > 11000	Hemoglobin	Hemoglobin < 11	Platelet	Platelet < 150k	C-reactive protein	Aspartate aminotransferase	Alanine aminotransferase
83	87	34.9	20	35	16380	1	6.9	1	72	1		445	269
80	104	36	26	105	11340	1	11.3	0	213	0	1.436	32	17
66	89	37.3	19	229	12270	1	11.7	0	119	1			
84	135	39.6	20	190	10670	0	10.1	1	103	1			
88	101	36.6	18	133	26590	1	11.5	0	255	0			15
75	83	36.4	20	n	10530	0	7.8	1			20.898		
59	95	36.9	20	80	10960	0	11	0	171	0	21.219		23
61	81	37.7	18	169	6580	0	11	0	161	0		97	102
55	88	36.8	18	226	5540	0	12.6	0	53	1	3.083	21	31
87	83	39.2	20	275	16420	1	11.9	0	206	0	19.009	16	25
97	110	38.7	22	113	9130	0	10.8	1	273	0	7.512		17
77	82	38.4	22	148	5820	0	10.9	1	175	0	2.432		
75	126	37.4	35	289	11210	1	7.4	1	459	0	3.027		
52	72	36	11	173	7880	0	9.2	1	422	0		16	
76	71	37.1	20	127	20230	1	10.1	1	275	0		20	13
61	121	36.4	26	154	15650	1	8.7	1	226	0	27.368	39	21
44	102	37	22	186	3890	1	11.4	0	193	0	10.123	18	11
74	90	36.4	20	122	11480	1	13.7	0	338	0		209	75
79	66	36.5	20	385	3240	1	13.6	0	203	0		8	12
81	98	36.3	17	101	3260	1	10.5	1	289	0		17	10
72	95	35.2	31	n	3520	1	11.9	0	78	1			
81	78	38	18	n	16500	1	8.1	1	396	0	3.376	20	56
76	67	37.5	20	112	10680	0	10.3	1	101	1	2.516		8
91	129	39.1	22	185	4130	0	9.3	1	54	1			79
69	103	37.4	22	102	7980	0	13.4	0	175	0	1.485		168
73	103	37.4	19	108	3760	1	8.6	1	69	1		310	140
95	118	37.5	20	92	14240	1	14.2	0	143	1	28.048	13	13
91	138	38.4	26	119	9100	0	14.4	0	170	0		52	24
50	102	37.5	27	126	12780	1	2.9	1	182	0	11.806	21	25
80	120	36	28	154	31250	1	10.9	1	446	0	5.346		30
70	91	38.4	32	n	11480	1	11.1	0	351	0	1.556	42	43
65	85	37	17	96	9590	0	9.3	1	160	0	7.535		
89	116	36.8	20	107	18360	1	12.1	0	325	0	15.621	26	33
66	100	37	20	n	13730	1	8	1	131	1	10.082		
70	80	37.7	20	n	2040	1	13.5	0	158	0	11.855	29	27
58	82	37	16	213	18010	1	8.6	1	359	0		19	35
60	90	36.3	22	245	15130	1	11	0	91	1	18.658	206	1271
50	68	36.8	20	n	8330	0	12.1	0	108	1		28	18
78	70	38.5	22	95	8940	0	11.1	0	137	1		27	10

74	66	39.1	22	101	8630	0	11.2	0	180	0	5.118		
52	118	37.6	22	139	5530	0	10.6	1	71	1	17.029	147	42
80	113	36.1	30	141	10720	0	12.9	0	214	0	7.811		20
85	98	38.8	16	111	12980	1	13.3	0	80	1	18.614	117	140
89	80	39	32	160	15680	1	8.9	1	148	1	10.951	48	
63	102	38.4	22	147	13920	1	8	1	236	0			37
83	96	39.4	22	161	11220	1	15.5	0	119	1	3.749	142	240
80	98	37.6	20	71	10680	0	8.5	1	155	0	18.495	14	6
72	113	38.1	19	136	7920	0	8.2	1	48	1	17.837	240	398
90	70	37.4	12	187	7420	0	9.1	1	185	0	0.667	24	28
54	109	37.5	14	176	8520	0	10.8	1	259	0		19	11
98	120	38.6	23	135	3270	1	9.8	1	214	0			7
48	108	37.2	29	119	21370	1	11.9	0	377	0	6.16	24	16
57	48	35.5	18	290	12420	1	13.9	0	178	0			
62	80	37.6	19	n	1980	1	8	1	137	1			
115	116	37	20	478	19160	1	13.8	0	328	0		47	19
82	106	36.6	20	219	8270	0	10.9	1	214	0	1.554	37	27
53	65	38	20	142	19300	1	13.7	0	106	1	10.749	30	17
123	105	35.2	20	173	7220	0	12.3	0	216	0		16	8
77	91	37	18	n	13350	1	9.2	1	77	1		84	21
98	82	37	20	119	4150	0	13.6	0	249	0		214	281
56	115	37.5	26	253	3160	1	10.4	1	249	0	22.995	43	7
73	96	37.6	24	118	7990	0	11.4	0	172	0	6.055		16
51	96	36.1	22	116	7690	0	9.5	1	209	0		37	
72	80	38.8	20	110	8420	0	11.2	0	371	0	3.834		54
82	67	37.6	22	135	5570	0	12.4	0	482	0	1.051		40
60	114	36.8	18	82	1360	1	6.7	1	271	0		41	24
82	82	37	20	186	7160	0	7.8	1	170	0		21	10
44	107	37.3	13	224	12320	1	10.3	1	121	1	27.538	31	10
68	95	38.3	24	n	3800	1	13.5	0	205	0			
61	65	37	18	97	4780	0	9.5	1	294	0		16	8
64	98	35.3	27	186	3420	1	9.3	1	113	1	19.938	37	25
71	69	36.2	21	210	6710	0	10.5	1	303	0			
55	67	36.8	13	131	7980	0	10.9	1	247	0		20	15
68	82	37.7	20	149	8670	0	10.3	1	154	0		19	18
80	133	39.5	30	127	6040	0	7.2	1	171	0		63	17
69	102	37.2	18	106	9640	0	10.8	1	98	1		56	30
128	100	38.4	24	n	10120	0	8.6	1	222	0	12.042	32	17
87	86	37.1	20	138	9360	0	12.7	0	175	0	3.728		16
61	66	37	18	n	4170	0	9.7	1	113	1	4.499	197	92
101	107	37	18	190	14900	1	11.1	0	330	0	4.61	22	25
68	116	37	22	201	11140	1	7.9	1	139	1	14.51	25	22

67	106	36	14	291	23640	1	8.5	1	102	1	0.651		
73	104	38.7	26	251	10780	0	8.1	1	50	1	15.861	28	23
112	113	36.6	22	188	10000	0	7.7	1	197	0			
49	81	38.2	20	n	16610	1	11.5	0	368	0	14.233	62	21
75	98	36.9	26	139	15380	1	10.2	1	38	1	2.012	141	156
57	128	37.2	28	381	13250	1	10.3	1	84	1			
91	76	39.6	20	n	12070	1	10.5	1	183	0	2.208	51	49
93	75	37.4	20	119	12330	1	8.4	1	342	0	12.958		
91	105	38.5	28	106	3020	1	11.4	0	106	1		51	30
71	60	36.7	23	125	17460	1	9.3	1	35	1			
65	93	35	20	117	9730	0	8.6	1	66	1	5.481		37
56	100	38.3	18	n	15760	1	9.2	1	365	0	36.689	67	16
69	77	36.6	20	n	5990	0	11.5	0	299	0	2.162	132	115
57	96	37.3	22	147	12490	1	13.1	0	291	0	0.28	31	26
106	91	37.2	20	n	9750	0	9.1	1	201	0			
76	100	37	22	210	3390	1	11.8	0	159	0	5.813	86	46
75	80	37.4	24	n	11790	1	11.2	0	197	0		52	41
40	101	36.6	22	444	9280	0	7.2	1	62	1			10
46	103	36.3	24	n	4510	0	7.2	1	90	1	3.714		22
78	100	39	22	137	21500	1	9.5	1	168	0	13.884	26	20
82	110	38	20	94	10510	0	10.9	1	154	0		19	8
67	74	36.4	22	126	42070	1	14.4	0	122	1	0.701	24	25
66	131	38.2	24	117	9380	0	12.3	0	250	0	0.052	30	25
72	94	36.1	22	352	23180	1	11.8	0	241	0	13.804	14	20
107	81	36.6	28	125	8090	0	10.6	1	228	0	6.559		698
61	101	37.5	26	110	11070	1	10.2	1	202	0	8.531	23	17
77	91	39	18	284	12700	1	11	0	95	1	1.755	37	43
94	111	37.6	24	134	12610	1	9.9	1	406	0	2.775	23	12
67	85	38.8	19	143	10640	0	13.7	0	166	0	21.128	22	22
90	124	38.6	36	149	10850	0	11	0	76	1	19.351		
54	80	36.9	23	203	15350	1	12.2	0	173	0	16.639	34	22
98	125	40.3	20	162	13360	1	12.2	0	368	0	3.438	26	21
58	74	35.9	20	124	12720	1	10.2	1	384	0		29	11
69	67	37	20	161	8150	0	11.8	0	221	0	6.415		13
66	78	37.1	20	133	15100	1	12.4	0	84	1	4.01	20	26
68	114	38.1	23	342	8650	0	8.2	1	147	1			
54	96	38.1	18	270	10890	0	10.5	1	231	0	23.004	21	19
52	81	35.9	20	48	6110	0	9.5	1	359	0	0.173	26	67
62	75	38.3	16	102	24210	1	11.3	0	219	0	20.405		29
58	85	37.3	23	80	6820	0	10.1	1	138	1			
70	100	37.5	19	93	3240	1	11.2	0	204	0	1.284	22	31
65	114	37.1	15	n	17270	1	8.3	1	180	0	10.527		

100	90	37.3	19	144	6670	0	8.6	1	65	1		15	5
81	86	37.3	20	N	3810	1	12.7	0	91	1	7.179	106	150
115	80	37.2	18	n	15900	1	9.5	1	100	1	2.162	37	31
70	130	37.9	26	206	8960	0	9.9	1	148	1	2.048	26	14
84	107	37.7	26	170	14280	1	13.4	0	317	0	5.343	13	8
95	83	36.5	20	116	9020	0	12.6	0	176	0		18	26
74	112	35.2	26	270	7590	0	14.5	0	235	0	1.674	20	13
102	100	38.4	20	140	11750	1	7.4	1	276	0		13	5
80	86	37.2	21	111	8170	0	11.9	0	40	1		147	175
70	110	36.4	18	323	7240	0	10.3	1	249	0			
65	107	38.3	24	N	9240	0	6.7	1	20	1	0.166	21	34
82	102	38.1	20	142	12810	1	15.3	0	108	1	33.784	35	29
56	74	36.4	22	117	4080	0	8	1	126	1	6.242	45	52
83	90	38.3	24	210	12610	1	12.5	0	430	0		19	19
86	82	36.5	20	191	11730	1	9.7	1	186	0		12	7
62	111	37.7	26	256	17830	1	7.5	1	287	0	8.49	24	21
77	111	38.5	29	207	14900	1	11.1	0	132	1		41	22
66	98	36.6	24	222	5340	0	10.2	1	376	0	10.334		
122	158	41.6	20	194	21010	1	15.3	0	203	0	5.318	31	30
52	92	37.2	18	136	7420	0	8.5	1	52	1		45	6
88	90	36.9	20	528	7560	0	14.2	0	130	1	27.623		27
70	74	38	20	258	14800	1	12.3	0	112	1	23.596	24	28
62	72	36.1	22	108	5940	0	10	1	202	0	4.985		
63	63	36.4	18	148	9830	0	9.3	1	345	0	5.1	22	28
61	102	37	22	277	21250	1	8.1	1	402	0	25.21		45
72	92	39.1	22	88	5920	0	9	1	313	0	4.227	35	23
43	144	37.2	23	n	6060	0	7.1	1	55	1			
53	120	37.3	29	98	8930	0	10.5	1	120	1	0.838	29	14
54	131	38.1	30	371	9280	0	10.8	1	235	0		36	12
67	84	38.6	24	103	14040	1	9.6	1	236	0	10.058	38	24
75	88	37.6	25	110	9880	0	12	0	211	0	1.188		
54	76	36	26	148	7310	0	9.8	1	205	0	11.885	11	13
78	131	41.1	28	n	9600	0	11.6	0	224	0	18.174	19	6
85	65	36	20	n	8190	0	10.3	1	207	0	1.486	16	6
66	87	36.8	20	208	6330	0	14.9	0	178	0			207
59	77	36	16	158	12770	1	7.5	1	62	1		26	29
54	94	37.7	94	n	8880	0	10.3	1	192	0		19	13
118	117	38.8	22	310	10040	0	30.6	0	390	0	4.516	27	18
94	104	36.8	20	783	18340	1	11.4	0	249	0		251	112
48	103	36	26	107	10190	0	9.7	1	227	0			48
73	100	38.5	20	438	16220	1	13.1	0	167	0	0.129	32	59
80	105	36.8	20	102	14450	1	12.3	0	382	0			10

Blood urea nitrogen	Creatinine	Creatinine > 1.4	Sodium	Potassium	Baseline Creatinine	Intensive care unit admission	Recommended dose	Duration of hospitalization	In-hospital mortality	time-to-onset of seizures (hours)	Computed tomography scan of brain	Electroencephalography
42.5	1.6	1	139	6.24	0.69	1	1	4	1	19.5	1	1
13.9	1.04	0	141	3.76	0.63	1	1	14	0			
			137	3.43	X	0	0	56	0			
22.7	1.08	0	137	4.14	0.75	0	1	23	0			
			138	4.24	X	0	0	7	0			
			131	3.03	7.48	1	0	41	0	38	1	1
23.4	0.81	0	136	4.28	0.5	1	1	19	0			
26	6.55	1	140	3.63	X	0	0	12	0			
22	0.78	0	137	3.57	0.65	0	1	33	0			
14.6	1.2	0	131	4.2	1.03	0	1	8	0			
22.9	1.3	0	134	3.5	0.61	0	1	10	0	45	1	0
10.5	1.07	0	147	2.69	0.7	0	1	19	0			
18.7	0.75	0	134	4.89	0.75	0	1	41	1			
96.5	3.51	1	140	2.76	1.23	1	0	19	0			
27.5	1.31	0	125	4.57	1.18	0	1	7	0			
			140	2.37	X	0	0	5	1	13	0	0
98.7	4.85	1	136	3.74	0.98	1	1	27	0			
11.8	0.83	0	136	3.1	0.69	0	1	20	0			
38.4	1.46	1	131	3.7	0.82	0	0	13	0			
111	9.96	1	127	5.58	0.78	1	0	27	0			
	1.22	0	149	4.43	1.04	1	1	23	1	51	0	1
54.1	2.63	1	141	4.43	2.25	0	0	20	0			
21	1.58	1	135	4.01	1.54	0	1	6	0			
16.1	0.86	0	129	2.85	0.83	0	1	10	0			
22.9	0.98	0	139	3.97	0.7	0	1	7	0			
72.4	6.07	1	141	3.69	5.89	0	0	19	1	189	1	1
38.1	1.86	1	136	4.09	1.66	0	1	11	0			
18.4	1.3	0	140	3.94	0.9	0	1	8	0			
18.7	0.97	0	122	4.73	0.58	1	1	14	0			
22.1	0.66	0	134	4.52	0.65	0	1	54	0			
26.1	0.96	0	134	4.17	0.78	0	1	41	0	56	0	1
15.7	0.67	0	142	3.11	0.67	1	1	15	0			
32.9	1.14	0	137	3.98	0.68	0	1	31	0			
	1.43	1	133	4.26	1.27	0	1	27	0			
20	1.13	0	131	4.29	0.95	0	1	12	0			
49.3	1.25	0	127	4.57	0.99	1	1	13	0	117.5	1	1
149.6	6.15	1	152	3	2.6	1	0	35	0			
22.4	1.09	0	128	3.01	1.04	0	1	32	0			
19.6	0.79	0	102	5.6	0.76	1	1	25	0			

11.2	1.06	0	131	4.09	0.86	0	1	18	0			
59.3	2.04	1	164	3.34	0.82	1	0	41	0	17	1	1
18.5	0.58	0	129	4.02	0.49	1	1	32	0			
23.4	1.2	0	140	3.46	1.06	0	1	25	0			
121.2	9.34	1	137	3.94	6.42	1	0	15	0			
79.9	2.1	1	138	4.11	1.22	0	0	18	0			
17.8	1.26	0	136	3.99	1.04	0	1	14	0	48	0	1
38.7	4.34	1	123	3.99	1.56	0	0	17	0			
27.1	1.45	1	139	3.1	1.08	1	1	21	0			
39.6	2.67	1	139	3.49	2.25	1	1	13	0			
18	1.53	1	131	3.62	0.9	0	1	17	0			
10.3	0.7	0	132	3.44	0.54	0	1	52	0	27	1	1
11.1	0.63	0	130	3.81	0.63	0	1	14	0			
12.2	1.11	0	146	3.76	0.87	1	1	17	0			
6.5	0.7	0	140	3.18	0.64	0	1	15	0			
12.6	0.82	0	129	5.12	0.82	0	1	12	0			
8.8	0.38	0	135	4.13	0.29	0	1	9	0	23	1, MRI	1
21.1	1.27	0	139	3.66	0.89	0	1	7	0			
28.7	5.95	1			X	0	0	7	0			
9.4	0.3	0	140	2.72	0.2	0	1	14	1			
11.8	0.47	0	132	4.1	0.36	0	1	52	1			
21.7	1.17	0	138	3.67	0.89	0	1	31	0	67.5	1	1
	1.04	0	137	3.09	0.99	0	1	10	0			
24.7	1.98	1	134	3.58	1.12	0	0	10	0			
19.4	1.12	0	136	3.9	0.85	0	1	10	0			
19.7	1.08	0	133	3.75	0.72	0	1	12	0			
77.6	3.37	1	137	4.27	2.06	0	0	19	0	75	1	1
54.4	1.65	1	136	5.4	1.39	0	0	10	0			
58.9	2.94	1	132	4.19	1.75	1	1	26	1			
20	0.28	0	140	3.08	0.28	0	1	18	0			
28.4	1.62	1	133	4.43	1.08	0	0	7	0			
40.5	1.2	0	99	4.6	0.8	0	0	6	1	69	0	1
	1.48	1	133	3.34	0.68	0	1	24	0			
47.9	1.86	1	137	3.68	0.94	1	0	13	0			
57.4	2.86	1	121	4.96	1.26	0	0	13	0			
38.1	0.85	0	134	4.71	0.52	0	1	54	1			
12.9	1.29	0	133	2.99	0.96	0	0	28	0	178	1	1
48.3	1.46	1	144	3.72	1.34	1	1	18	0			
21.1	1.06	0	137	3.95	0.59	0	1	12	0			
13.7	1.03	0	135	4.4	0.99	0	1	15	0			
25.9	1.61	1	127	4.68	1.17	0	0	11	0			
106	2.93	1	140	4.29	1.87	0	1	7	1	16	1	0

139.3	5.49	1	132	3.07	4.2	1	0	24	0			
30.7	1.67	1	137	4.22	1	0	1	6	0			
					X	1	0	35	0			
14.5	1.08	0	143	4.38	0.81	0	1	16	0			
146.7	4.73	1	135	4.36	4.43	1	0	5	1	55.5	0	0
13.9	0.87	0	134	4.17	0.83	0	1	59	1			
12.8	1.24	0	135	4.34	1.05	0	1	11	0			
12.1	1.52	1	137	3.35	1.1	0	1	25	0			
9.3	1.35	0	139	3.43	1.05	1	1	12	0			
18.3	1.03	0	131	2.92	0.51	1	1	15	1	65	0	0
54.8	5.2	1	132	2.79	3.09	0	1	28	1			
					X	0	0	17	0			
19.9	1.03	0	136	3.18	0.84	0	1	25	0			
39	1.5	1	138	3.73	0.93	0	0	20	0			
53.8	2	1	130	4.08	1.47	0	0	20	0	67.5	0	0
35.7	1.2	0	129	3.74	0.78	0	0	17	0			
13.1	0.54	0	141	2.94	0.52	1	1	17	0			
89.6	1.85	1	151	3.89	0.79	1	0	24	0			
11.2	1	0	138	4	0.95	0	1	15	0			
18	1	0	133	2.73	0.7	0	1	53	0	114	1	1
25.7	1.75	1	139	3.98	1.28	0	0	13	0			
21.9	1.23	0	140	4.51	1.14	0	1	8	0			
16.3	1.36	0	140	3.78	1.23	0	1	5	1			
24.8	1.69	1	132	4.04	1.39	0	1	17	0			
10.5	0.76	0	107	3.87	0.7	1	1	15	0	261	0	0
27.8	1.12	0	137	3.67	0.88	0	1	11	0			
26.3	1.19	0	131	3.72	1.12	0	1	8	0			
73.5	6.13	1	137	3.13	6.09	1	1	24	0			
			142	4.35	X	0	0	11	1			
25.4	1.72	1	148	2.9	0.75	0	0	30	1	249	1	1
32.8	1.72	1	158	2.75	1	1	0	21	0			
16.8	1.09	0	135	3.91	0.96	0	1	10	0			
13.7	1.24	0	128	3.01	0.9	1	1	13	0			
9.8	0.54	0	137	3.58	0.33	0	1	9	0			
14.7	0.86	0	139	3.58	0.59	0	1	70	0	71.5	1	1
20.4	0.74	0	154	3.56	0.56	1	1	29	1			
77.1	2.22	1	134	3.38	1.84	1	1	12	0			
23.8	0.8	0	139	3.97	0.8	0	1	11	0			
17.5	1.35	0	130	4.42	1.17	0	1	67	0			
11.1	0.71	0	135	3.62	0.64	1	1	64	0	90	1	1
11.6	0.62	0	135	4.27	0.46	0	1	10	0			
14	0.61	0	142	3.06	0.54	1	1	45	1			

63.3	5.14	1	135	4.13	5.14	0	1	17	0			
24.2	1.15	0	138	4.06	0.93	0	1	20	0			
41.2	2.31	1	143	3.11	2.09	0	0	26	1	9	0	0
30.4	2.05	1	139	4.17	1.61	0	1	10	0			
37.4	2.98	1	145	3.28	1.61	0	0	9	0			
18	1.04	0	137	3.27	0.79	0	1	7	0			
19.8	0.73	0	128	2.76	0.58	1	1	10	0			
94	11.33	1	137	4.18	10.53	0	0	14	0	35.5	1	1
32.2	1.25	0	138	3.98	0.78	0	1	9	0			
15.2	0.26	0	124	4.67	0.26	0	1	38	0			
51.3	0.72	0	153	3.21	0.46	0	1	18	1			
55.8	3.69	1	133	3.34	1.18	0	0	11	0			
39	0.55	0	118	3.33	0.47	0	1	14	1	137.5	0	1
15.7	1.01	0	137	3.11	0.76	0	1	29	0			
50.5	5.12	1	132	4.58	5.02	0	1	10	0			
37.3	2.92	1	129	3.1	1.91	0	0	23	0			
35.8	3.29	1	139	3.22	1.17	1	1	28	0			
27.8	0.81	0	153	2.4	0.49	0	1	25	0	44	0	0
11.8	0.95	0	138	3.74	0.81	0	1	8	0			
116.9	4.8	1	130	4.84	3.51	1	0	13	0			
23.4	0.62	0	129	4.73	0.42	1	1	34	0			
60.9	2.93	1	137	4.15	1.58	0	1	19	0			
8.2	0.4	0	138	3.49	0.36	0	1	14	0	102	0	1
21	1.01	0	135	3.95	0.86	0	1	35	0			
33.1	1.13	0	133	3.72	0.94	0	1	15	0			
17.6	0.37	0	129	4.24	0.36	0	1	10	0			
71.2	0.87	0	153	4.1	0.85	1	1	41	1			
56.5	0.97	0	154	3.27	0.66	1	1	32	0	104	0	1
40.9	1.67	1	138	2.84	0.86	1	1	53	1			
68.8	2.31	1	134	4.54	1.73	0	0	11	0			
24.5	0.62	0	133	4.47	0.56	0	0	33	0			
9.4	1.04	0	128	3.31	0.74	0	1	13	0			
11.8	1.36	0	143	4.22	0.63	0	1	25	0	121.5	1	1
40.5	5	1	139	2.79	2.09	0	0	11	0			
9.7	0.82	0	137	3.76	0.77	0	1	6	0			
191.7	9.39	1	153	3.92	3.26	1	1	36	0			
20.7	1.02	0	135	3.58	0.82	0	1	12	0			
23.7	1.17	0	131	4.21	0.9	0	0	15	0	29	0	1
95.2	1.68	1	142	3.77	1.49	1	1	9	0			
22.7	0.79	0	143	3.47	0.66	1	1	17	0			
58.3	1.42	1	161	4.16	0.6	0	1	11	0			
33.5	1.54	1	131	2.74	1.29	0	1	15	0			

Medications for control of seizures Benzodiazepines	Medications for control of seizuresAntiepileptic drugs	previous drug history within one month before admission	Calcium channel blocker	ACEI or ARB	Diuretics	Beta blocker	Alpha blocker	Harnalidge	Nitrate	Statin	Antibiotics	H2-blocker
1	1	1	1	0	1	0	0	0	0	0	1	1
		0	0	0	0	0	0	0	0	0	0	0
		0	0	0	0	0	0	0	0	0	0	0
		1	0	0	0	0	0	0	0	0	0	1
		1	1	0	0	0	0	0	0	0	0	0
1	1	1	1	0	0	0	0	0	0	0	0	0
		1	0	0	0	0	0	0	0	0	0	0
		1	1	0	0	0	0	0	0	0	0	0
		1	0	0	1	0	0	0	0	0	0	0
		0	0	0	0	0	0	0	0	0	0	0
0	1	1	0	0	0	0	0	0	0	0	0	0
		1	0	0	0	0	0	0	0	0	0	1
		1	0	1	0	0	0	0	0	0	0	0
		1	0	0	0	0	0	0	0	0	0	0
		0	0	0	0	0	0	0	0	0	0	0
1	1	1	0	0	0	0	0	0	0	0	0	1
		1	1	0	0	0	0	0	0	0	0	0
		1	1	1	1	1	0	0	0	0	0	1
		1	1	0	0	1	0	0	0	0	0	1
		1	1	1	1	1	0	0	1	0	1	1
1	1	1	0	0	0	0	0	1	0	0	0	0
		1	1	1	0	0	1	0	0	0	0	0
		0	0	0	0	0	0	0	0	0	0	0
		1	1	1	1	0	1	0	0	0	0	0
		0	0	0	0	0	0	0	0	0	0	0
0	1	1	1	0	0	0	1	0	0	1	0	1
		1	0	0	0	0	0	0	0	0	0	0
		0	0	0	0	0	0	0	0	0	0	0
		1	0	0	0	0	0	0	0	0	1	1
		0	0	0	0	0	0	0	0	0	0	0
0	0	1	0	0	0	0	0	0	0	0	0	0
		0	0	0	0	0	0	0	0	0	0	0
		0	0	0	0	0	0	0	0	0	0	0
		1	1	0	0	0	0	0	0	0	0	0
		1	1	1	1	1	0	0	0	1	0	1
1	1	1	0	1	1	1	0	0	0	0	0	0
		1	0	0	1	1	0	1	1	0	0	1
		0	0	0	0	0	0	0	0	0	0	0
		1	0	0	0	0	0	0	0	0	0	0

		1	0	0	0	0	0	0	0	0	1	0
0	1	1	1	0	0	0	0	0	0	0	0	1
		1	1	0	0	0	0	0	0	0	0	0
		0	0	0	0	0	0	0	0	0	0	0
		0	0	0	0	0	0	0	0	0	0	0
		1	0	0	1	0	0	0	0	0	0	0
1	1	0	0	0	0	0	0	0	0	0	0	0
		1	1	0	0	0	0	1	0	0	0	0
		1	0	0	1	0	0	0	0	0	0	1
		1	1	0	0	0	1	0	1	0	1	0
		1	0	0	0	0	0	0	0	0	0	0
1	1	1	1	1	0	0	0	0	0	0	0	0
		1	1	0	0	0	0	0	0	0	0	0
		0	0	0	0	0	0	0	0	0	0	0
		0	0	0	0	0	0	0	0	0	0	0
		0	0	0	0	0	0	0	0	0	0	0
1	1	1	1	1	1	0	0	0	0	1	1	0
		0	0	0	0	0	0	0	0	0	0	0
		1	0	0	0	0	0	0	0	0	1	0
		1	1	0	0	0	0	0	0	0	0	1
		1	0	0	0	0	0	0	0	0	0	0
1	1	1	1	0	0	0	0	0	0	0	0	0
		1	1	0	0	0	0	0	0	0	0	0
		1	0	0	0	0	0	0	1	1	0	0
		0	0	0	0	0	0	0	0	0	0	0
		1	1	0	0	0	0	1	1	0	1	1
1	0	0	0	0	0	0	0	0	0	0	0	0
		1	0	0	0	1	0	0	0	0	0	0
		1	1	1	1	0	0	0	0	0	0	0
		1	0	0	0	0	0	0	0	0	0	1
		1	0	0	0	1	0	0	0	0	1	1
0	1	0	0	0	0	0	0	0	0	0	0	0
		1	0	0	1	1	0	0	1	0	0	1
		1	0	1	0	0	0	0	0	0	1	0
		1	1	1	1	0	0	0	0	1	0	0
		1	0	0	0	1	0	0	0	0	0	1
1	1	1	0	0	0	0	0	0	0	0	0	1
		1	1	1	0	0	0	0	1	0	0	1
		1	1	0	0	0	0	0	0	0	0	0
		1	0	0	0	0	0	0	0	0	1	1
		1	1	0	1	0	0	0	0	0	1	0
1	1	1	0	0	0	0	0	0	0	0	0	0

		0	0	0	0	0	0	0	0	0	0	0
		1	0	0	0	0	0	0	0	0	0	0
		1	1	1	1	0	0	0	0	1	0	1
		0	0	0	0	0	0	0	0	0	0	0
1	1	1	0	0	0	0	0	0	0	0	1	0
		1	0	0	0	0	0	0	0	0	0	0
		1	0	0	0	0	0	0	0	0	1	0
		1	0	0	0	0	0	0	0	1	0	0
		1	0	0	0	0	0	0	0	0	0	0
1	1	1	1	0	0	0	0	0	0	0	0	0
		1	0	0	1	0	0	0	0	0	0	1
		0	0	0	0	0	0	0	0	0	0	0
		1	1	0	0	0	0	0	1	0	0	1
		1	0	0	0	1	1	0	0	0	0	0
1	1	1	1	0	1	0	0	0	0	0	0	0
		1	1	1	0	0	0	0	0	0	0	1
		0	0	0	0	0	0	0	0	0	0	0
		0	0	0	0	0	0	0	0	0	0	0
		1	0	0	0	0	0	0	0	0	1	0
0	1	1	1	0	0	0	0	0	0	0	1	1
		1	0	0	0	0	0	0	0	0	1	1
		1	1	1	1	0	0	0	0	0	0	0
		1	1	0	0	0	0	0	0	0	0	0
		1	1	1	0	0	1	0	0	0	0	0
0	1	1	0	0	0	0	0	0	0	0	1	1
		0	0	0	0	0	0	0	0	0	0	0
		1	0	0	0	0	0	0	0	0	0	0
		1	1	1	0	1	0	0	0	0	0	1
		1	0	0	0	0	0	0	0	0	0	1
1	1	1	0	0	0	0	0	0	0	0	0	1
		1	0	0	1	0	0	0	0	0	0	0
		1	0	0	0	0	0	0	0	0	0	1
		1	0	0	0	0	0	0	0	0	1	0
		1	1	0	0	0	0	1	0	0	0	1
0	1	1	1	0	0	0	0	1	1	0	0	0
		1	1	0	0	0	0	0	0	0	0	0
		0	0	0	0	0	0	0	0	0	0	0
		1	0	0	1	0	0	0	0	0	0	0
		1	1	0	0	1	0	1	0	0	0	0
1	1	1	0	0	0	0	0	1	0	0	0	0
		1	0	0	0	0	0	1	0	0	0	1
		1	0	0	0	0	0	1	0	0	1	0

		1	1	1	1	0	0	0	0	0	0	0
		0	0	0	0	0	0	0	0	0	0	0
1	1	1	1	0	1	0	0	0	0	0	1	1
		1	1	0	1	0	0	1	0	0	0	1
		1	0	0	0	0	0	0	0	0	0	0
		1	0	0	0	0	0	1	0	0	0	0
		1	0	0	1	0	0	0	0	0	0	0
1	1	1	0	0	0	0	0	0	0	0	0	0
		0	0	0	0	0	0	0	0	0	0	0
		0	0	0	0	0	0	0	0	0	0	0
		1	0	0	0	0	0	1	0	0	0	0
		1	0	0	0	0	0	1	0	0	1	1
1	1	1	1	0	0	0	0	0	0	0	0	0
		1	0	1	1	0	0	0	1	1	0	0
		1	1	0	0	0	0	0	0	1	0	0
		0	0	0	0	0	0	0	0	0	0	0
		1	1	1	0	1	1	1	0	0	0	1
0	0	1	1	1	1	1	0	0	0	1	1	0
		1	1	0	0	0	1	0	0	0	1	0
		1	0	0	0	1	0	0	0	1	0	0
		0	0	0	0	0	0	0	0	0	0	0
		0	0	0	0	0	0	0	0	0	0	0
0	1	1	0	0	0	0	0	0	0	0	0	0
		1	0	0	0	0	0	0	0	0	1	0
		1	0	0	0	0	0	0	0	0	1	1
		1	1	1	0	0	0	0	0	0	0	0
		1	0	0	0	0	0	0	0	0	0	1
1	1	0	0	0	0	0	0	0	0	0	0	0
		1	0	1	1	1	1	0	0	0	0	0
		1	1	1	0	0	1	0	0	0	0	0
		1	1	0	1	0	1	0	0	0	0	1
		1	0	0	1	0	0	0	0	0	0	0
1	1	1	1	0	0	0	0	1	0	0	0	0
		0	0	0	0	0	0	0	0	0	0	0
		1	0	1	0	0	0	0	0	1	0	0
		1	0	0	0	0	0	0	0	0	0	0
		0	0	0	0	0	0	0	0	0	0	0
1	1	1	0	0	0	0	0	0	0	0	0	0
		1	1	0	1	0	0	0	1	0	0	0
		0	0	0	0	0	0	0	0	0	0	0
		1	1	0	0	0	0	0	0	0	0	0
		1	1	0	1	0	0	0	0	0	0	0

Proton pump inhibitors	Steroid	Antiplatelet agents	Prokinetic agents	Drugs for peripheral vascular disorder	Sedative/hypnotics	Concomitant drugs with Invanz	Proton pump inhibitors	H2-blocker	Prokinetic agents	Harnalidge	Albumin	Opioids	Non-steroid anti-inflammatory drug
0	0	0	0	0	0	1	1	0	0	0	1	0	0
0	0	0	0	0	0	1	0	0	1	0	0	0	0
0	0	0	0	0	0	1	1	0	0	0	0	1	0
0	0	0	1	0	0	1	1	0	0	0	0	1	1
0	0	1	0	0	0	1	1	1	0	0	0	0	0
0	0	1	0	1	0	1	1	0	0	0	0	1	0
0	1	0	1	0	1	1	0	1	0	0	1	1	0
0	0	1	0	0	0	1	0	1	1	0	1	1	0
0	0	0	0	0	0	1	1	0	0	0	1	0	0
0	0	0	0	0	0	1	0	0	1	0	0	0	0
0	0	0	1	0	0	1	0	0	1	0	0	0	0
0	0	0	1	0	0	1	0	1	1	0	0	0	0
0	0	1	0	1	1	1	1	1	1	1	0	0	0
0	0	0	0	0	0	1	0	1	1	0	1	0	0
0	0	0	0	0	0	1	1	0	0	0	0	0	0
0	1	0	0	1	0	1	1	1	0	0	0	0	0
0	0	0	0	0	0	1	1	0	1	0	1	1	0
0	0	0	1	0	0	1	0	1	1	0	0	0	0
0	0	0	0	0	0	1	0	1	0	0	0	0	0
1	1	1	0	0	0	1	1	0	0	0	0	1	0
0	0	0	0	0	0	1	1	0	0	1	0	0	0
0	0	0	0	1	0	1	1	0	0	1	0	0	0
0	0	0	0	0	0	1	0	0	0	0	0	0	1
1	0	0	0	0	0	1	0	0	0	0	0	1	1
0	0	0	0	0	0	1	0	0	0	0	0	0	0
0	0	0	1	1	0	1	0	1	1	0	0	0	0
0	0	1	0	0	0	1	1	1	1	0	1	1	0
0	0	0	0	0	0	1	0	0	0	1	0	0	0
1	0	0	0	0	1	1	1	0	0	0	1	0	0
0	0	0	0	0	0	1	0	1	0	0	0	0	0
0	0	0	0	0	0	1	0	1	1	0	0	0	0
0	0	0	0	0	0	1	0	1	1	0	0	0	0
0	0	0	0	0	0	1	1	0	1	1	0	0	1
1	1	0	1	0	0	1	1	0	0	0	0	0	0
0	0	0	0	0	1	1	0	1	0	0	0	1	0
0	1	0	0	0	0	1	0	0	0	0	0	0	0
0	1	1	0	1	0	1	1	0	0	0	0	0	0
0	0	0	0	0	0	1	0	0	1	0	0	0	0
0	0	0	1	1	0	1	0	0	0	0	1	0	0

1	0	0	0	0	0	1	1	0	0	0	0	0	0
0	0	1	0	0	0	1	0	0	0	0	0	0	0
0	0	0	0	0	0	1	0	1	0	0	1	1	0
0	0	0	0	0	0	1	0	0	0	1	1	0	0
0	0	0	0	0	0	1	0	0	0	0	0	0	0
0	1	0	0	0	1	1	0	1	1	0	1	0	0
0	0	0	0	0	0	1	0	0	0	0	0	1	0
1	0	0	0	0	1	1	0	0	0	1	0	1	0
0	1	0	0	0	0	1	1	1	1	0	0	1	0
0	0	1	0	0	0	1	1	1	0	0	0	0	0
1	0	0	0	0	0	1	1	0	0	0	1	0	0
0	0	1	0	0	0	1	1	0	0	0	0	0	0
0	0	1	0	0	0	1	0	0	0	0	0	0	0
0	0	0	0	0	0	1	1	0	1	0	1	1	0
0	0	0	0	0	0	1	1	0	1	0	1	0	0
0	0	0	0	0	0	1	0	1	1	0	0	1	0
1	0	1	0	0	0	1	1	0	0	0	0	0	0
0	0	0	0	0	0	0	0	0	0	0	0	0	0
1	0	0	1	0	0	1	1	0	1	0	0	0	0
0	0	0	0	1	1	1	0	1	0	0	1	1	0
0	0	0	0	0	0	1	1	0	1	0	0	1	1
0	0	0	1	0	0	1	0	0	1	0	0	0	0
0	0	0	0	0	1	1	0	0	0	0	0	0	1
1	0	1	1	0	0	1	1	0	1	0	0	0	0
0	0	0	0	0	0	1	1	1	0	0	0	1	0
0	0	1	0	0	0	1	0	1	0	1	0	0	0
0	0	0	0	0	0	1	1	1	0	0	1	0	0
0	0	0	0	0	0	1	0	0	0	0	0	0	0
0	1	0	0	1	0	1	1	0	0	0	1	1	0
0	0	1	1	1	1	1	0	1	0	0	0	0	0
0	0	0	0	0	0	1	0	1	0	0	0	0	0
0	0	0	0	0	0	1	0	0	1	0	0	0	0
0	0	1	0	0	0	1	1	0	0	0	0	0	0
0	0	1	0	0	1	1	0	1	1	0	0	0	0
0	0	1	0	0	0	1	0	1	0	0	1	1	0
0	1	0	1	1	0	1	1	0	0	0	0	0	0
0	0	0	0	0	0	1	1	0	1	0	1	0	0
0	0	0	1	0	0	1	0	0	0	0	0	0	0
0	1	1	0	1	0	1	0	0	0	0	0	0	0
0	0	0	1	0	1	1	0	1	1	0	0	0	0
1	0	1	1	0	1	1	0	0	1	0	0	0	0
0	0	0	0	0	0	1	1	0	1	0	0	1	0

0	0	0	0	0	0	1	1	0	1	0	1	0	0
0	0	0	0	0	1	1	0	0	0	0	0	1	0
0	0	0	0	0	0	1	0	1	1	0	0	0	0
0	0	0	0	0	0	1	1	1	1	0	1	0	0
1	1	0	0	0	0	1	1	0	0	0	0	1	0
0	1	1	0	0	0	1	0	0	0	0	1	1	0
0	0	0	1	0	1	1	0	1	1	0	1	0	0
0	0	0	0	0	1	1	1	0	1	0	0	0	0
0	0	0	0	0	0	1	1	1	1	0	0	1	0
0	0	0	0	0	0	1	0	1	0	0	0	0	0
0	0	0	1	1	1	1	1	1	0	1	1	1	0
0	0	0	0	0	0	0	0	0	0	0	0	0	0
0	0	1	1	0	1	1	1	1	0	0	0	1	0
0	0	1	1	0	0	1	0	0	1	0	0	0	0
0	0	0	0	0	1	1	1	0	0	0	0	0	0
0	0	0	0	0	1	1	0	0	1	0	0	0	0
0	0	0	0	0	0	1	0	1	0	0	0	0	0
0	0	0	0	0	0	1	0	1	1	0	1	1	0
1	0	0	0	0	0	1	1	0	0	0	0	0	1
0	0	0	0	0	0	1	0	1	1	0	0	0	0
1	0	0	0	0	0	1	0	0	0	0	0	0	0
0	0	1	0	0	0	1	0	0	0	0	0	0	0
0	0	0	0	0	0	1	0	1	0	0	0	0	0
0	0	0	0	0	0	1	0	1	0	0	0	0	0
1	0	0	0	0	0	1	1	1	1	0	1	1	0
0	0	0	0	0	0	1	0	0	0	1	0	0	0
0	1	0	0	0	0	1	0	0	1	0	0	0	0
0	0	0	1	1	1	1	0	1	0	0	0	0	0
0	0	0	0	0	0	1	1	0	0	0	0	1	0
0	0	0	1	0	0	1	1	1	1	0	0	0	0
0	1	0	1	0	0	1	0	0	1	0	0	0	0
1	0	1	1	0	0	1	1	0	1	0	0	0	0
0	0	0	0	0	1	1	0	0	0	0	0	0	0
0	0	0	0	0	0	1	0	1	0	1	0	0	0
0	0	0	1	0	0	1	1	1	0	1	0	0	0
0	0	0	1	0	1	1	1	0	0	0	1	0	0
0	0	0	0	0	0	1	1	1	0	0	1	0	0
0	0	0	0	0	1	1	0	1	0	1	0	0	0
1	0	0	0	0	0	1	1	0	0	1	0	1	0
0	0	0	0	0	0	1	0	0	0	0	0	0	0
0	0	0	0	0	0	1	1	0	1	0	0	0	0
1	0	0	1	0	1	1	1	0	0	0	0	0	0

1	0	1	1	0	1	1	0	0	0	0	0	0	0
0	0	0	0	0	0	1	0	1	0	0	0	0	0
0	0	0	1	0	0	1	0	0	1	0	0	0	0
0	0	0	0	0	0	1	0	1	0	0	0	0	0
1	1	0	0	0	0	1	0	0	1	0	0	1	0
0	1	0	0	0	0	1	0	0	1	1	0	0	0
0	0	0	0	0	0	1	0	1	0	0	0	0	0
1	0	0	1	0	0	1	0	0	0	0	0	0	0
0	0	0	0	0	0	1	1	0	0	0	0	0	0
0	0	0	0	0	0	1	0	0	0	0	0	0	0
1	0	0	1	0	0	1	1	0	0	0	1	0	0
0	0	0	0	0	0	1	0	0	0	1	0	0	0
0	0	0	1	0	1	1	1	0	0	0	0	0	0
1	0	1	0	0	0	1	1	0	0	0	0	0	0
1	0	1	1	1	1	1	1	0	0	0	0	0	0
0	0	0	0	0	0	1	0	0	0	0	0	0	0
0	0	0	1	0	1	1	0	0	0	0	0	0	0
0	0	1	1	0	0	1	1	0	1	0	0	1	0
0	0	1	0	0	0	1	0	0	0	0	0	0	0
0	0	1	0	1	0	1	0	0	0	0	0	0	0
0	0	0	0	0	0	1	1	0	0	0	0	1	0
0	0	0	0	0	0	1	0	1	1	0	0	0	0
0	0	0	0	0	0	1	0	1	0	0	0	0	0
0	0	0	0	0	0	1	0	0	0	0	0	0	1
0	1	1	1	1	0	1	0	1	1	0	0	0	0
0	0	0	0	1	0	1	0	0	0	0	0	0	0
0	1	0	0	0	0	1	0	1	1	0	0	1	0
0	0	0	0	0	0	1	0	1	0	0	1	0	0
1	0	1	0	0	0	1	1	0	0	0	0	0	0
1	0	0	0	0	0	1	1	0	0	0	0	0	0
0	1	1	0	0	0	1	0	0	0	0	0	0	0
0	0	0	0	1	0	1	1	0	0	0	0	0	0
0	0	0	0	0	0	1	1	0	0	0	1	0	0
0	0	0	0	0	0	1	0	0	0	1	0	0	0
0	0	1	0	0	1	1	0	0	0	0	0	0	1
0	0	0	1	0	0	1	1	1	0	0	1	0	0
0	0	0	0	0	0	1	0	1	0	0	0	0	0
0	0	0	0	0	0	1	0	0	0	0	0	0	0
0	0	0	0	0	0	1	1	1	0	0	0	1	0
0	0	0	0	0	0	1	0	1	0	0	0	1	0
0	0	1	0	0	0	1	0	0	0	0	0	0	0
1	0	0	1	0	1	1	1	0	0	0	0	0	0

Steroid	Inhaled bronchodilator	Beta-blocker	Diuretics	Calcium channel blockers	Statin	Nitrate	Cordarone	Knowful	Antiplatelet agents	Drugs for peripheral vascular disorder	Transamine	Sedative/hypnotics	Antibiotics	Contrast medium
0	1	0	1	0	0	0	0	0	0	0	0	0	0	0
1	0	0	0	1	0	0	0	0	0	0	0	0	0	1
0	0	0	0	1	0	0	0	0	0	0	0	0	1	0
0	0	0	0	0	0	0	0	0	0	0	0	0	0	0
0	0	0	0	0	0	0	0	0	0	0	0	0	0	1
0	1	0	0	0	0	0	0	0	0	0	0	1	0	0
1	0	0	1	0	0	0	0	0	0	0	1	0	1	0
0	1	0	0	0	0	0	0	0	0	0	1	1	0	0
0	0	0	1	0	0	0	0	0	0	0	0	0	0	0
0	0	0	0	0	0	0	0	0	0	0	0	0	0	0
0	0	0	0	0	0	0	0	0	0	0	0	0	0	0
0	1	0	1	1	0	0	0	0	0	0	0	0	1	0
1	1	0	0	1	0	0	0	0	0	1	0	0	1	1
0	0	0	1	0	0	1	0	0	1	0	0	0	1	0
0	0	0	0	0	0	0	0	0	0	0	0	0	1	0
0	0	0	0	0	0	0	0	0	0	0	0	0	0	0
0	1	0	1	0	0	0	0	0	0	0	1	1	0	1
0	0	0	0	1	0	0	0	0	0	0	0	0	0	0
0	0	1	0	1	0	0	1	1	0	0	0	0	0	0
1	0	1	1	1	0	0	0	0	0	0	0	0	0	0
0	0	1	0	1	0	0	0	0	0	0	1	0	1	0
0	0	0	1	0	0	0	0	0	0	0	0	0	0	0
0	0	0	0	1	0	0	0	1	1	0	0	0	0	0
0	1	0	0	0	0	0	0	0	0	0	0	0	0	0
0	1	0	0	0	0	0	0	0	0	0	1	0	0	1
0	0	0	0	1	1	0	0	0	0	1	1	0	0	0
0	1	0	0	0	0	0	0	0	1	0	1	0	0	1
1	1	0	0	0	0	0	0	0	0	0	0	1	0	0
0	0	0	1	0	0	0	0	0	1	1	0	0	0	1
1	0	0	1	0	0	0	1	0	1	0	0	0	0	0
0	0	0	1	0	0	0	0	0	0	0	0	0	0	0
0	0	0	1	1	0	0	0	0	0	0	0	0	0	0
0	1	0	0	0	0	0	0	0	0	0	0	0	1	1
1	1	0	0	0	0	0	0	0	0	0	0	0	0	0
1	1	0	0	1	1	0	0	0	0	0	0	1	0	0
0	1	1	0	0	0	0	0	0	0	0	0	0	0	0
0	1	0	1	0	0	1	0	0	0	0	0	0	0	0
0	0	0	0	0	0	0	0	0	0	0	0	0	0	0
0	1	0	1	0	0	0	0	1	0	0	0	0	1	0

0	0	0	0	0	0	0	0	0	0	0	0	0	1	1
0	0	0	0	0	0	0	0	0	0	0	0	0	0	0
1	1	0	1	1	0	0	0	0	0	0	1	1	0	0
0	0	0	0	0	0	0	0	0	0	0	0	0	0	0
1	0	1	1	1	0	0	0	0	0	1	0	0	1	0
1	0	0	1	0	0	0	0	0	0	0	0	0	0	0
0	0	0	0	0	0	0	0	0	0	0	0	0	0	1
0	0	1	0	1	0	0	0	0	0	0	0	1	0	0
1	1	0	0	0	0	0	0	0	0	0	0	0	0	1
1	0	1	1	1	0	1	0	0	0	0	0	0	0	0
0	0	0	0	0	0	0	0	0	0	0	0	0	1	0
0	0	0	0	1	0	0	0	0	1	0	0	0	1	0
0	1	0	0	0	0	0	0	0	1	0	0	0	0	0
0	1	0	1	1	0	0	1	0	0	0	1	0	0	0
0	1	0	0	0	0	0	0	0	0	0	1	0	0	0
0	1	0	0	0	0	0	0	0	0	0	0	1	0	1
0	0	0	0	1	1	0	0	0	1	0	0	0	0	0
0	0	0	0	0	0	0	0	0	0	0	0	0	0	0
0	0	0	0	0	0	0	0	0	0	0	0	0	1	0
0	0	0	0	0	0	0	0	0	0	0	0	0	0	0
0	0	0	0	0	0	0	0	0	0	0	0	0	0	0
1	1	0	1	1	0	0	0	0	0	0	0	1	0	0
0	1	0	0	1	0	0	0	0	0	0	0	1	0	0
1	0	0	0	0	1	0	0	0	1	0	0	1	0	0
0	0	0	0	0	0	0	0	0	0	0	0	0	0	1
0	0	0	0	1	0	1	0	1	1	0	1	0	0	0
0	0	0	1	0	0	0	0	0	0	0	0	0	1	0
1	0	1	0	1	0	0	0	1	0	0	0	0	0	0
0	0	0	1	1	0	0	0	0	0	0	1	0	0	0
1	0	0	1	1	0	0	0	1	1	0	1	0	0	0
0	0	1	0	0	0	0	1	0	0	0	1	0	0	0
0	1	0	0	0	0	0	0	0	0	0	0	0	0	0
0	0	1	1	0	0	1	1	0	0	0	0	0	0	0
0	0	0	0	1	0	0	0	0	1	0	0	0	0	0
1	1	0	1	1	1	0	0	0	1	0	0	0	1	0
1	1	0	1	1	0	0	0	0	0	1	1	0	1	0
0	0	0	0	0	0	0	0	0	0	0	0	0	0	0
0	0	1	1	1	0	1	0	1	1	0	0	0	0	0
1	1	0	0	0	0	0	0	0	0	0	0	0	1	0
0	0	0	0	0	0	0	0	0	0	0	0	1	0	0
0	0	1	1	1	0	0	0	1	1	0	0	1	0	0
0	1	0	1	0	0	1	0	0	0	0	1	0	0	0

1	0	0	1	0	0	0	0	0	0	0	0	0	1	0
1	1	0	1	0	0	0	0	0	0	0	1	1	0	0
0	0	1	0	1	0	1	0	0	1	0	0	0	0	0
1	0	0	0	0	0	0	0	0	0	0	0	0	0	0
0	0	0	1	0	0	0	0	0	0	0	0	0	0	0
1	1	0	1	0	0	0	0	0	0	0	0	0	0	0
0	0	1	0	1	0	0	0	0	0	0	0	1	0	0
1	0	0	0	1	1	0	0	0	0	0	0	1	0	0
0	0	0	1	1	0	0	0	0	0	0	1	1	1	0
1	1	0	0	0	0	0	0	1	0	0	0	1	0	0
0	0	0	1	0	0	0	0	0	0	0	0	0	0	0
0	0	0	0	0	0	0	0	0	0	0	0	0	0	0
1	0	1	1	1	0	0	0	0	1	0	0	0	0	0
0	1	1	1	1	0	0	0	0	1	0	0	0	0	0
0	0	0	1	0	0	1	0	0	0	0	0	0	0	0
0	0	0	1	0	0	0	0	1	0	0	0	1	0	0
1	1	0	0	1	0	0	0	0	0	0	0	0	0	0
0	0	0	1	0	0	0	0	0	0	1	0	0	0	0
0	0	0	0	1	0	0	0	0	0	0	0	1	0	0
0	1	0	0	1	0	0	0	1	0	0	1	0	0	0
0	0	0	0	0	0	0	0	0	0	0	0	1	0	0
1	0	1	0	0	0	0	0	0	1	0	0	1	0	0
1	1	0	1	1	0	0	0	0	0	0	0	0	0	0
1	0	0	0	1	0	0	0	0	0	0	0	1	0	0
1	1	0	1	0	0	0	0	1	0	0	1	0	0	1
0	1	0	0	0	0	0	0	0	1	0	0	0	0	0
0	0	0	1	0	0	0	0	0	0	0	0	0	0	1
1	1	0	0	1	0	0	0	0	0	0	0	0	0	0
0	0	0	0	0	0	0	0	0	0	0	1	0	0	0
0	1	0	0	1	0	0	1	0	1	0	0	1	1	0
1	1	0	1	0	0	0	0	0	0	0	0	0	1	0
0	0	0	0	0	0	0	0	0	1	0	0	0	0	0
1	0	0	0	0	0	0	0	0	0	0	0	1	1	1
0	0	0	0	1	0	0	0	0	0	0	0	0	0	0
1	1	0	0	1	0	1	0	0	0	0	0	0	0	0
0	1	0	1	1	0	0	0	0	0	0	0	1	1	1
0	1	0	0	0	0	0	1	0	0	0	1	0	0	0
0	1	0	0	0	0	0	0	1	0	0	0	0	0	0
0	0	1	1	1	0	0	0	0	0	0	0	0	1	0
0	0	0	0	0	0	0	0	1	0	0	0	1	0	0
0	1	0	0	0	0	0	0	0	0	0	0	0	0	0
0	0	0	1	1	0	0	1	0	0	0	0	0	0	0

0	0	0	0	0	0	0	0	0	0	0	0	0	0	0
0	1	0	0	0	0	0	0	0	0	0	0	0	0	0
0	0	0	1	0	0	0	0	0	0	0	1	0	0	0
1	1	0	1	1	0	0	0	0	0	0	0	0	0	0
1	0	0	0	1	0	1	0	0	0	0	0	0	1	0
1	0	0	0	0	0	0	0	0	0	1	0	0	0	0
0	0	0	0	0	0	0	0	0	1	0	0	0	0	0
0	0	0	0	0	0	0	0	0	0	0	0	0	0	0
0	0	0	0	0	0	0	0	0	0	0	0	0	0	0
0	1	0	0	0	0	0	0	0	0	0	0	0	1	0
0	0	0	1	0	0	0	0	0	0	0	0	0	1	1
0	0	0	0	0	0	0	0	0	0	0	0	0	0	0
0	1	0	0	0	0	0	0	0	0	0	1	0	1	0
0	0	0	1	0	0	1	1	0	1	0	0	0	1	0
0	0	0	0	1	1	0	0	0	1	0	0	0	1	0
0	1	0	0	0	0	0	0	0	0	0	0	0	0	0
0	0	0	0	0	0	0	0	0	0	0	0	1	1	0
0	0	0	0	0	0	0	0	0	0	1	0	0	0	0
0	1	0	0	1	0	0	0	0	1	0	0	0	0	0
0	0	1	0	0	1	0	0	0	1	1	0	0	0	0
0	0	0	0	0	0	0	0	0	0	0	1	1	0	1
0	1	0	0	1	0	0	0	0	0	0	0	0	0	0
0	0	0	0	0	0	0	0	0	0	0	0	0	0	0
0	0	0	0	0	0	0	0	0	1	1	0	1	0	0
1	1	0	0	0	0	0	0	0	0	0	0	0	1	0
0	1	0	0	1	0	0	0	0	0	0	0	0	0	0
1	0	0	1	0	0	0	1	0	0	0	0	0	1	0
0	1	0	1	0	0	0	0	0	0	0	0	0	1	0
1	1	0	1	1	0	0	1	0	0	0	0	0	1	0
0	0	0	0	1	0	0	0	0	0	0	0	0	0	0
1	0	0	1	1	0	1	0	0	1	0	0	0	0	0
0	1	0	0	0	0	0	0	0	0	0	0	0	0	0
0	1	0	0	1	0	0	0	0	0	0	0	0	0	0
0	0	0	0	1	0	0	0	0	0	0	0	0	0	0
0	0	0	0	0	0	0	0	0	0	0	0	0	0	1
0	1	0	0	0	0	0	0	0	0	0	0	0	0	0
0	0	1	0	0	0	0	0	0	1	0	0	0	1	1
0	0	0	0	1	0	0	0	0	0	0	0	0	0	0
0	0	0	0	0	0	0	0	0	0	0	0	0	0	0
1	0	0	0	0	0	0	0	0	0	0	0	0	1	1
0	1	0	0	0	0	0	0	0	1	0	0	0	0	0
1	0	0	1	1	0	0	0	0	0	0	0	0	0	0
